# Magnesium-organophosphate bone adhesives repurposed as endodontic cements for dental applications

**DOI:** 10.1007/s00784-026-06743-9

**Published:** 2026-02-04

**Authors:** Tobias Renner, Laura Marx, Niclas Fleckenstein, Valentin C. Steinacker, Paul F. Otto, Alexander C. Kübler, Uwe Gbureck

**Affiliations:** 1 Department for Functional Materials in Medicine and Dentistry, University Hospital Würzburg, Pleicherwall 2, 97070 Würzburg, Germany; 2 Department of Oral & Maxillofacial Plastic Surgery, Head and Neck Surgery, University Hospital Würzburg, Pleicherwall 2, 97070 Würzburg, Germany

**Keywords:** Mineral organic cement, Adhesive dental material, Temporary restoration, Dye penetration test, Bone adhesive

## Abstract

**Objectives:**

This study investigated novel mineral-organic cements based on magnesium and organophosphates, originally developed as resorbable bone adhesives. The aim was to evaluate this new class of materials in a dental context, focusing on their suitability as temporary restorative materials in endodontic treatments.

**Materials and methods:**

Two thermally treated trimagnesium phosphate hydrates (Mg₃(PO₄)₂ ∙ xH₂O and Mg₃(PO₄)₂ ∙ 22H₂O (cattiite)) were each combined with two organophosphates (phosphoserine (OPLS) and sodium phytate (Na-IP6)), resulting in four adhesive formulations. These were compared with a commonly used material for temporary restorations (Cavit™) and a reference adhesive (TTCP + OPLS) regarding mechanical strength, sealing ability (methylene blue penetration) on bovine teeth, and structural integrity.

**Results:**

Shear bond strength to dental hard tissues was significantly higher for the new cements compared to Cavit™, particularly for the OPLS + TMP ∙ xH₂O formulation (4.35 ± 0.71 MPa vs. 0.29 ± 0.16 MPa; initially on dentin). pH and temperature during setting remained within clinical limits. TMP ∙ xH₂O + OPLS also showed the favorable sealing behavior, while Cavit™ performed similarly. Porosimetry, SEM and micro-CT supported the favorable profile of this formulation.

**Conclusion:**

The combination of 400 °C sintered TMP ∙ xH₂O and OPLS proved most suitable in terms of handling, stability, and sealing. This system are promising candidates for temporary restorations, particularly in non-retentive cavities.

**Clinical relevance:**

The tested cements may serve as novel resorbable, adhesive alternatives for temporary restorations. Future applications could include pulp capping or perforation repair.

**Graphical abstract:**

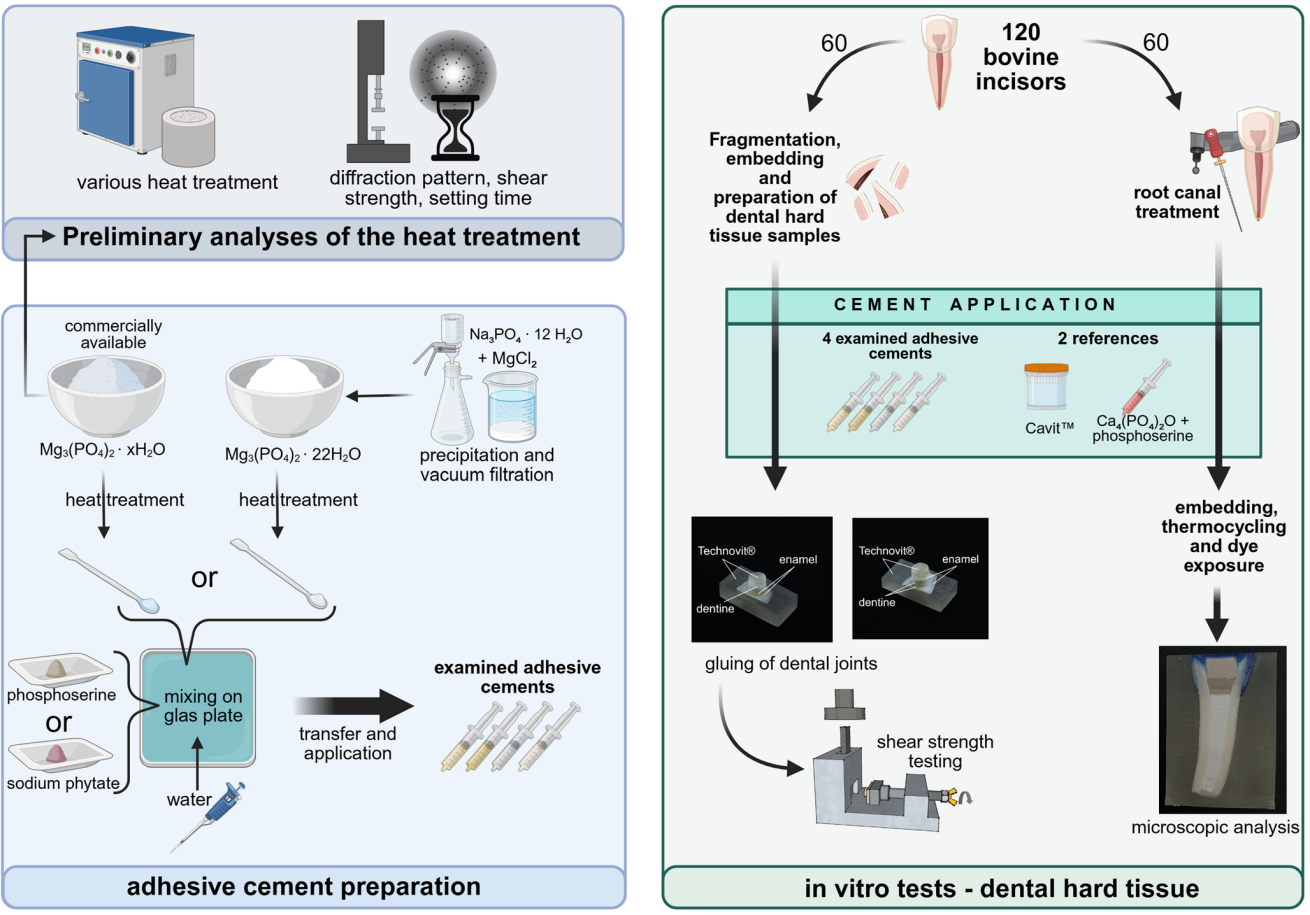

**Supplementary Information:**

The online version contains supplementary material available at 10.1007/s00784-026-06743-9.

## Introduction

A key objective of endodontic filling materials, for both temporary and permanent applications, is to achieve a bacteria-tight seal of the root canal system, thereby preventing bacterial re-infection and ensuring long-term clinical success [1]. This fundamental requirement forms the basis for evaluating any new material class intended for endodontic use. In the search for novel endodontic materials that meet these sealing and handling requirements, attention has increasingly turned to material classes originally developed for other medical disciplines. One particularly promising example is the recent development of mineral-organic bone adhesives. These materials, which combine bioactivity with strong adhesion to mineralized tissues, offer properties that could also be highly relevant for endodontic applications.

Bone adhesives are emerging as promising tools for surgical bone fixation and regeneration. Despite extensive research, no adhesive has yet reached widespread clinical adoption. Over the past decade, significant efforts have focused on developing “mineral-organic”, bioresorbable bone adhesives that meet both clinical handling requirements and biocompatibility standards. Early in vitro studies by Reinstorf et al. [2] demonstrated low cytotoxicity and favorable cell compatibility for phosphoserine-modified calcium phosphate cements, which later informed the development of mineral-organic bone adhesives. A major breakthrough was achieved by Kirillova et al. [3], who introduced a bioinspired calcium phosphate-phosphoserine-based adhesive, marking the first generation of such materials. Foley et al. [4] successfully applied this adhesive for cranial flap fixation in a sheep model, while Cochran et al. [5, 6] evaluated its effectiveness for dental implant stabilization in oversized osteotomies. Hulsart-Billström et al. [7] further confirmed the in vivo biocompatibility and safety profile in a dedicated preclinical safety study. Geddes et al. [8] provided additional biomechanical evidence for fracture stabilization in dogs, while Sugita et al. [9] reported radiographic and histological support for osseointegration and long-term bone maintenance using this novel bone adhesives around implants in canine oversized osteotomies. Translating these findings to the clinical setting, Norton [10] conducted a pilot human study, demonstrating the technical feasibility and safety of adhesive use during immediate implant placement. Subsequently, Pikos and Miron [11] published the first detailed human case report with long-term follow-up, documenting three years of complication-free healing without local or systemic toxicity. Together, this cumulative evidence—from early in vitro compatibility testing, through targeted adhesive design and extensive animal studies, to first clinical applications—highlights mineral-organic resorbable bone adhesives as a biocompatible, mechanically reliable, and clinically promising alternative to conventional fixation techniques across multiple surgical disciplines.

However, despite these promising results, challenges such as reliable curing under physiological conditions and further improvement of material properties remain. Recent research has therefore explored alternative mineral phases. In this context, magnesium-based systems have gained attention. Magnesium plays a critical role in osteogenesis, enhances bone remodeling, and exhibits favorable degradation behavior compared to calcium-based systems [12–14]. Studies indicate that magnesium phosphate cements may outperform their calcium phosphate counterparts in various respects, including faster setting times [13], superior mechanical strength [14], and improved biological responses such as enhanced osteoconductivity and cell proliferation [12]. Biomineral adhesive cements containing magnesium oxides or phosphates instead of calcium compounds have shown increased bond strength to mineralized substrates in previous studies [15]. Especially the use of amorphous magnesium phosphate in the context of these adhesives offers improved water stability [15]. These novel cements, combining TMP hydrates and phosphoserine, have demonstrated superior adhesion values compared to other leading bone adhesive candidates [15]. The described material group of adhesive cements is particularly noteworthy due to its magnesium base, which exhibits a strong affinity for organophosphates. To further optimize the adhesive performance of the developed magnesium-based cements, sodium phytate was investigated as a novel component in this study. Its potential contribution to adhesive strength and biological compatibility is supported by prior studies on phytic acid, the conjugate acid of sodium phytate, in bone adhesives [16, 17].

The emerging class of adhesive cements based on magnesium compounds and organophosphates—such as phosphoserine or phytic acid derivatives—represents more than just a solution for bone repair. Due to their strong bonding to mineralized tissues, bioactivity, controlled degradation, and biocompatibility, these materials provide a versatile platform for a wide range of medical and dental applications. Given their potential for dental applications, it is worth noting that dentistry has historically played a pioneering role in the development and clinical adoption of medical cements. Especially in this field, dentistry is regarded as a driving scientific force. Materials such as PMMA, for example, which were first established in dentistry, sometimes found their way into other disciplines later [18]. In the case of the biomineral adhesive bone cements presented here, however, the process could be reversed: a bone adhesive originally intended for surgical use may now find application in dentistry. This assumption appears reasonable, as hydroxyapatite—the primary inorganic component of bone—is also a major constituent of tooth enamel and dentin [19]. The adhesive properties of the cements described above are probably due to chelate complexes with the calcium of the inorganic bone phase [15], and similar mechanisms may also apply to dental hard tissues.

In this context, endodontics represents a particularly promising field of application. One specific example is the use of adhesive cements as temporary restorative fillings during root canal treatments. These procedures are typically performed in multiple stages, requiring interim fillings that provide both effective sealing and easy retrievability. Conventional temporary filling materials lack adhesive properties and often require a retentive cavity design to stay in place [1]. This can result in unnecessary loss of tooth structure or an increased risk of filling loss during treatment intervals. Traditional dental adhesives, on the other hand, are time-consuming to apply and often difficult to remove. Cements, including adhesive mineral-organic cements described here, could address these challenges by offering reliable sealing without the need for additional mechanical retention. Their lower adhesive strength compared to bonded composites allows for easy removal, especially with the aid of ultrasound, while their material composition ensures clear distinction from the surrounding tooth structure during removal. Furthermore, these cements are typically cost-effective, making them an economically attractive option for temporary restorations in endodontic therapy.

The topic of “endodontic cover fillings” serves as a foundational example for assessing adhesion to dental tissue and microleakage. Moreover, these materials hold potential for various dental applications, including covering cervical perforations, pulp capping, and apexification, among others.

 The primary objective of this study is to investigate, for the first time, the application of a novel class of biomineral cement-like substances, composed of magnesium compounds and organophosphates, originally developed for bone applications, in dental procedures. Specifically, this study focuses on their use as provisional endodontic cover fillings. Therefore, it examines whether the new adhesive (bone) cements provide improved bond strength to teeth compared to a conventional material (Cavit™) and whether they achieve a comparable level of sealing effectiveness.

## Materials and methods

### Raw powder syntheses, cement syntheses and system analyses

Commercially available, multiphase Mg₃(PO₄)₂ ∙ xH₂O (with an undefined hydration state, Thermo Fisher Scientific Inc., Waltham, USA) was sintered at 400 °C in a high-temperature furnace (Nabertherm GmbH, Lilienthal, Germany) prior to cement synthesis. For analytical purposes, additional samples were heat-treated at temperatures ranging from 50 °C to 800 °C in 50 °C increments. To serve as reference, the untreated starting material (i.e., without any heat treatment) was included in the analyses and labeled as “0°C” in figures and data presentations. The furnace was heated to 100 °C within 200 min (at ≤ 100 °C directly), this temperature was maintained for 30 min and then increased to the target temperature within a further 200 min. After six hours of sintering, the material cooled down to room temperature in the furnace. Finally, the heat-treated powder was sieved to < 355 μm.

As a structurally well-defined reference phase, cattiite was prepared by a precipitation reaction of 50 ml 0.4 M Na₃PO₄ ∙ 12 H₂O and 50 ml 0.6 M MgCl₂ (both Sigma-Aldrich GmbH, Steinheim, Germany). The solutions were prepared, mixed in a 1:1 ratio and vacuum-filtered with the addition of acetone and ultrapure water. After drying at 37 °C overnight, the phase purity was confirmed by XRD analysis. Sintering was carried out according to the protocol described above at 400 °C, the powder was sieved to < 355 μm and the amorphousness was verified by XRD. To prepare the raw TTCP powder, 12.1 mol CaHPO₄ was homogenized with 11.5 mol CaCO₃ (both Merck KGaA, Darmstadt, Germany) in a ploughshare mixer for 60 min, sintered at 1500 °C for five hours and then sieved to < 125 μm.

The powders prepared as above were combined with phosphoserine or sodium phytate (both Sigma-Aldrich GmbH, Steinheim, Germany) with the addition of water. The resulting materials used for the coronal cover filling are summarized in Table 1. These formulations are based on earlier adhesive magnesium–organophosphate cements introduced by Renner et al. (2023) [15], and were further developed prior to this study. Their compositional optimization and biological characterization in bone-related contexts were carried out independently of the present work, which focuses on their dental application.

**Table 1:** Materials to be tested and their composition

labelling	composition		PLR
(1)	Na-IP6	330 mg	3,53
	Commercial TMP hydrate (Mg_3_(PO_4_)_2_ ∙ xH_2_O, heat-treated)	200 mg	
	H_2_O	150 µl	
(2)	Na-IP6	510 mg	2,96
	Cattiite (Mg_3_(PO_4_)_2_ ∙ 22H_2_O, heat-treated)	200 mg	
	H_2_O	240 µl	
(3)	OPLS	160 mg	2,33
	Commercial TMP hydrate (Mg_3_(PO_4_)_2_ ∙ xH_2_O, heat-treated)	400 mg	
	H_2_O	240 µl	
(4)	OPLS	240 mg	2,13
	Cattiite (Mg_3_(PO_4_)_2_ ∙ 22H_2_O, heat-treated)	400 mg	
	H_2_O	300 µl	
(5) (reference)	Cavit™ (3 M ESPE AG, Landsberg am Lech, Germany)		
(6) (reference)	OPLS	150 mg	4,23
	TTCP (Ca_4_(PO_4_)_2_O)	400 mg	
	H_2_O	130 µl	

**Fig. 1:**
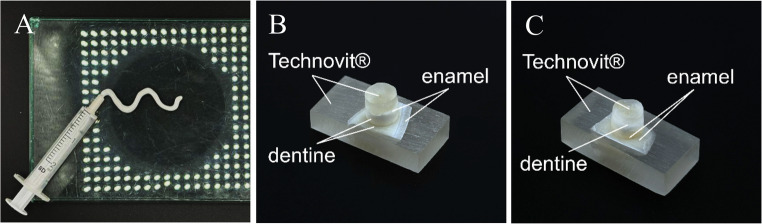
Exemplary illustrations of (**A**) the applicability of one of the cement compositions presented using a 2 ml syringe, (**B**) bonded dentin test specimens and (**C**) bonded enamel test specimens

The novel cements were mixed on a glass plate with the aid of a spatula while being continuously spread against the glass plate. After transferring to a 2 ml syringe, the cements could be applied. Processing was completed within 1 min. The application is shown as an example in Fig. 1A.

Initial powders and synthesized cements were measured with the XRD D8 Advance (Bruker Corporation, Billerica, United States) using Cu K-α radiation, an accelerating voltage of 40 kV and an emission current of 40 mA. The diffraction angle was 2 θ. It was plotted from 10 ° to 70 ° with 1.2 s per 0.02 °. Files from the database of the International Center of Diffraction Data (PDF-2, 1996) were used as reference samples. The open porosity was determined using the porosimeter PASCAL 140/440 (Porotec GmbH, Hofheim, Germany) with a surface tension of the mercury γ = 480 mN/mm and a diffraction angle of θ = 141.3 °. The range of measurable pore sizes extends from 4 nm to 0.4 mm.

Temperature developments during the setting mechanism were measured over a period of 20 min using the Voltcraft^®^ K202 temperature measuring device with thermal sensor (Conrad Electronic SE, Hirschau, Germany). The change in pH during the setting reaction was measured using an InLab™ surface pH electrode (Mettler-Toledo International Inc., Columbus, United States) and an InoLab Level 2 pH meter (WTW GmbH, Weilheim, Germany).

### Sample preparation, mechanical testing and fracture analysis

For testing the shear bond strength on various surfaces (hydroxyapatite, dentin/Technovite^®^, enamel/Technovite^®^) specimens of the several parts to be bonded were created as described in the following.

Prior to hydroxyapatite sample production, molds were created using addition-cured vinyl polysiloxane Dublisil^®^ (Dreve Dentamid GmbH, Unna, Germany). Prefabricated molds with cubic raised features (20 mm x 10 mm x 5 mm) and cylindrical raised features (π x (2.5 mm)² x 5 mm) were utilized to shape the specimens. Dublisil^®^ components were mixed in a 1:1 ratio, poured into the molds, and left to set for 45 min. Once cured, Dublisil^®^ impressions were demolded and used to produce identical hydroxyapatite cubes and cylinders. All other cuboid samples to be bonded mentioned below, whether enamel or dentin, had the above-mentioned dimensions with regard to their surface area.

Hydroxyapatite samples were explicitly used only for initial analyses of the effect of sintering temperature on bond strength and setting speed (see Fig. 3, results) in order to have more standardized conditions. It was synthesized from α-tricalcium phosphate (α-TCP) and 2.5% Na_2_HPO_4_. 0.716 mol CaHPO_4_ (Lot: H1630; Honeywell, USA) and 0.33 mol CaCO_3_ (Lot: A0915620609; Merck, Darmstadt, Germany) were mixed and sintered at 1400 °C. The resulting α-TCP powder (< 355 μm) was dry-milled for 4 h. 3 g of α-TCP powder was mixed with 1 ml 2.5% Na_2_HPO_4_, and the paste was used to fill Dublisil^®^ molds. Filled molds were cured at 37 °C and 100% humidity in a TW20 water bath (Julabo GmbH, Seelbach, Germany) for 7 days. Excess water was removed in a drying oven (Memmert GmbH & Co. KG, Schwabach, Germany) before use. Surfaces were roughened with P80 SiC wet abrasive paper (Schmitz Metallographie GmbH, Herzogenrath, Germany) prior to bonding. X-ray diffraction analyses were conducted on all synthesis components.

Bovine incisors were obtained from the slaughterhouse (Henke GmbH, Feuchtwangen, Germany; Rinderfarm Hochrein Angus, Eisenheim, Germany). Incisor segments were sawed off, clamped, and extracted using a lever and incisor forceps. The root was freed from the desmodont with a scalpel and the tooth was preserved in 0.5% chloramine-T solution at 5 °C until use. Enamel and dentin fragments were extracted using a diamond-coated separator in a dental handpiece (ULTIMATE 1:5, B.A. International GmbH, Hamburg, Germany). Fragments were embedded in Technovit^®^ 4004 (Kulzer GmbH, Hanau, Germany), roughened with P80 SiC wet abrasive paper, and used for bonding. The tooth test specimen production process is shown in more detail in Fig. 11 (appendix A.1). 12 enamel and 12 dentin cylinders were required per bonded cement composition and per measurement time (*n* = 12). The dental hard substance cylinders were stored in a simple stored at 5 °C in a simple PBS solution until bonding. Figure 1 (B, C) shows examples of bonded dentin and enamel test specimens.

The prospective bonding surfaces on the test specimens were manually roughened with SiC wet abrasive paper grain P80 and the surfaces were cleaned with compressed air. The samples were stored at 37 °C in PBS before bonding. Tooth test specimens were removed from the liquid directly before bonding, dabbed with a paper towel and stored for 1 h at 25 °C in a drying chamber which could correspond to the conditions with a rubber dam in place. Rijke et al. or van Pelt et al. also chose a temperature of 25 °C [20, 21]. Immediately before bonding, compressed air was applied for 20 s at distances of 1–2 cm. The new cements were mixed as described above. Any excess cement was carefully removed with a spatula. Once glued, the specimens were then stored in a TW20 water bath (Julabo GmbH, Seelbach, Germany) at 37 °C and 100% humidity until the end of the setting process. 12 blocks were bonded with corresponding cylinders (*n* = 12) for each bonded material (enamel, dentin, hydroxyapatite) and for each time unit. Measurements were taken initially and after 1 h, 24 h and 7 d. In addition to the ZwickRoell Z010 universal testing machine (ZwickRoell GmbH & Co. KG, Ulm, Germany), a custom-built shear testing device allowing controlled lateral loading of the bonded interface was used, as described previously [15, 22] and schematically illustrated in the Supplementary Material (Fig. S1). A 10 kN load cell was used for all tests. Measurements were taken from a preload of 1 N, whereby the test speed was 1 mm/min. The test was automatically aborted at a drop in force of 80% of the maximum measured force.

Shear bond strength data were analyzed using one-way or two-way ANOVA, depending on the experimental setup. For enamel, a two-way ANOVA was used to evaluate the effects of material and storage time; for dentin, one-way ANOVA was applied at each time point. Tukey’s HSD test was used for post-hoc comparisons. Statistical significance was defined as *p* < 0.05.

The remaining adhesive joints on the joined parts were examined after shearing using the Zeiss Crossbeam 340 scanning electron microscope (Carl Zeiss AG, Oberkochen, Germany). Prior to this, the samples were coated with 4.0 nm platinum at a pressure of 10^− 7^ mbar on the EM ACE600 sputter coater (Leica Camera, Wetzlar, Germany). The scanning electron micrographs, including secondary electrons, were taken at 3.00 kV with a vacuum of 1.25 × 10^− 6^ and an aperture of 30.00 μm.

In addition to the adhesive strength, the compressive strength of the materials was also tested. For this purpose, 10 cement blocks measuring 12 mm x 6 mm x 6 mm were produced for each material (*n* = 10) using the above-mentioned Dublisil^®^ moulages and stored in a water bath at 37 °C and 100% humidity until testing after the above-mentioned times. The test was automatically aborted at a drop in force of 80% of the maximum measured force or a deformation of 20%.

### Tooth preparation for dye penetration test

**Fig. 2:**
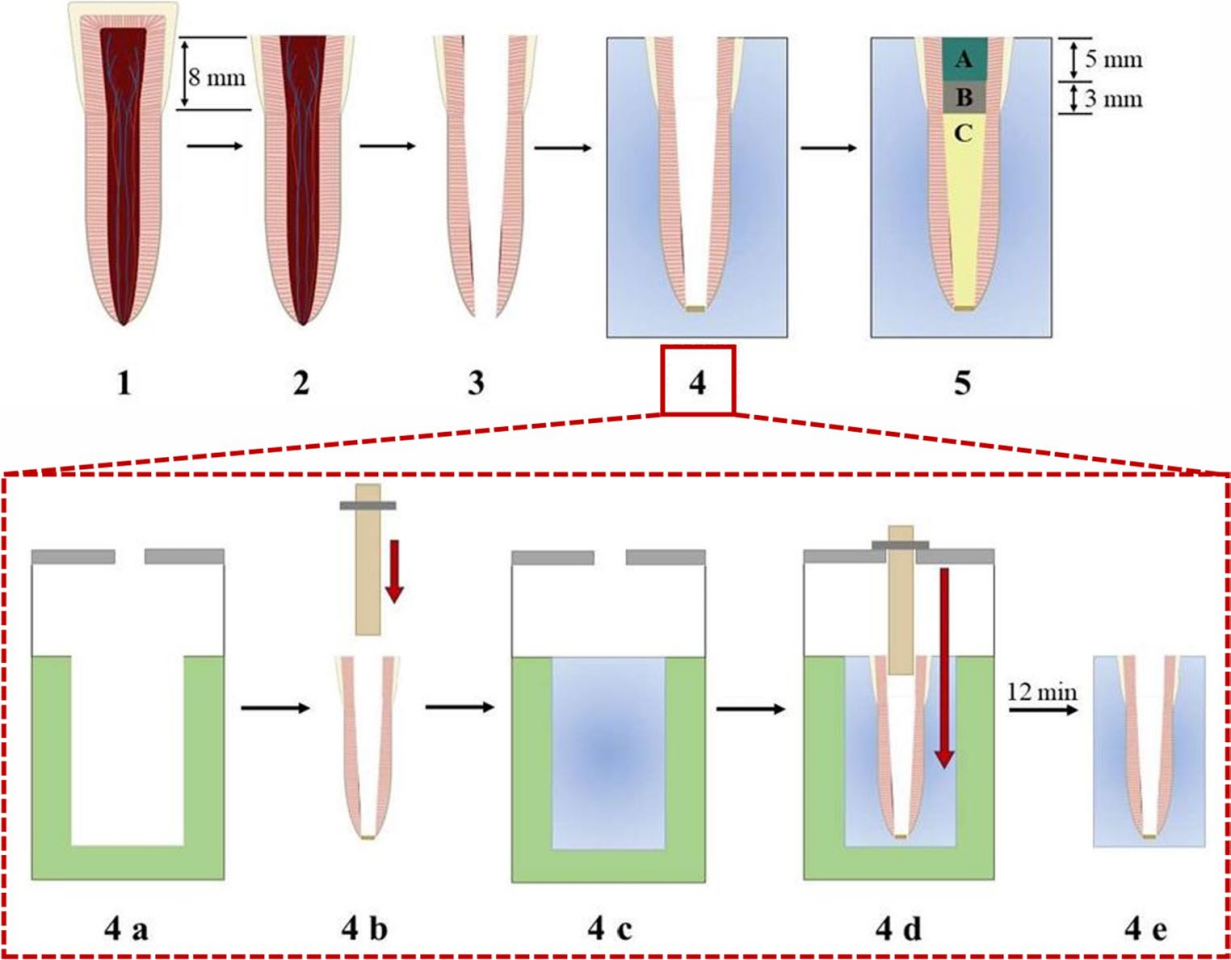
Preparation steps including embedding and root canal treatment of the bovine anterior teeth for evaluation of sealing performance. (1) Extracted and cleaned bovine incisor. (2) Crown reduced to a residual height of 8 mm. (3) Removal of pulp tissue and mechanical preparation of the root canal. (4) Embedding procedure using Technovit® 4004: **a**) Silicone mold with mounted holder for tooth fixation; **b**) Stabilizing device inserted into the pulp cavity, apex sealed with composite; **c**) Mold filled with Technovit® 4004; **d**) Tooth suspended and fixed in resin before curing; **e**) Final test body after polymerization and demolding. (5) Root canal filling (**A**: Investigated adhesive cement or Cavit™ as reference; **B**: Cavit™ G; **C**: Ca(OH)₂)

The individual steps for the production of the endodontically treated bovine anterior test specimens can be seen in Fig. 2.

A total of 60 anterior teeth were used for the dye penetration test, which resulted in 12 samples per tested cement. A standardized residual crown height of 8 mm was determined. The portion of the crown that was not required was removed with a contra-angle handpiece. The anterograde trepanation was performed with a diamond-coated roller. The access cavity was widened and all overhangs and tissue remnants of the pulp cavity were removed. The root canal system was prepared with K-files (VDW GmbH, Munich, Germany) up to ISO 110 while rinsing with ultrapure water. Finally, it was rinsed again and dried. The apical foramen was sealed with Scotchbond Universal Plus Adhesive (3 M, Minnesota, United States) and Tetric EvoFlow A1 (Ivoclar Vivadent AG, Schaan, Liechtenstein) and light-cured. The prepared anterior teeth were placed in moulages made of Dublisil^®^ with a recess measuring 25 mm x 25 mm x 35 mm using a suspension (see Fig. 2) and embedded in Technovit^®^ 4004 while protecting the root canal system. The junction between the resin and the exposed tooth surface was leveled and smoothed on the disk grinder with SiC wet abrasive paper grit P80 under water cooling. The root canal was filled up to the enamel-cement junction with a paste of Ca(OH)_2_ and NaCl. Coronally to this, all samples were equally filled with Cavit™ G (3 M ESPE AG, Landsberg am Lech, Germany). The residual height of 5 mm was then filled with the respective materials to be tested.

After overnight curing of the cover fillings to be examined, the embedded teeth were subjected to thermocycling to simulate an intraoral ageing process over 21 days. The thermocycling (LAUDA Dr. R. Wobser GmbH & Co. KG, Lauda Königshofen, Germany) was carried out by alternately placing the samples in a 5 °C and a 55 °C water bath. The duration of each cycle was 1 min with 30 s per basin. A total of 210 cycles were run. The teeth were then immediately immersed in a 2% methylene blue solution (pure methylene blue, Carl Roth GmbH & Co. KG, Karlsruhe, Germany) and left to soak on the Rotamax 120 platform shaker (Heidolph Instruments GmbH & Co. KG, Schwabach, Germany) at 50 rpm for 7 days. Finally, the samples were prepared from labial to oral up to the center of the anterior tooth using SiC wet-grinding paper on the Metaserv^®^ 3000 disk grinder so that the filling and root canal system could be assessed in longitudinal section. The bulk dye penetration including interfacial dye microleakage with methylene blue was assessed under the Leica TL5000 Ergo light microscope. Figure 6 (results) defines the extent of the interfacial leakage and the bulk dye penetration.

## Results

**Fig. 3:**
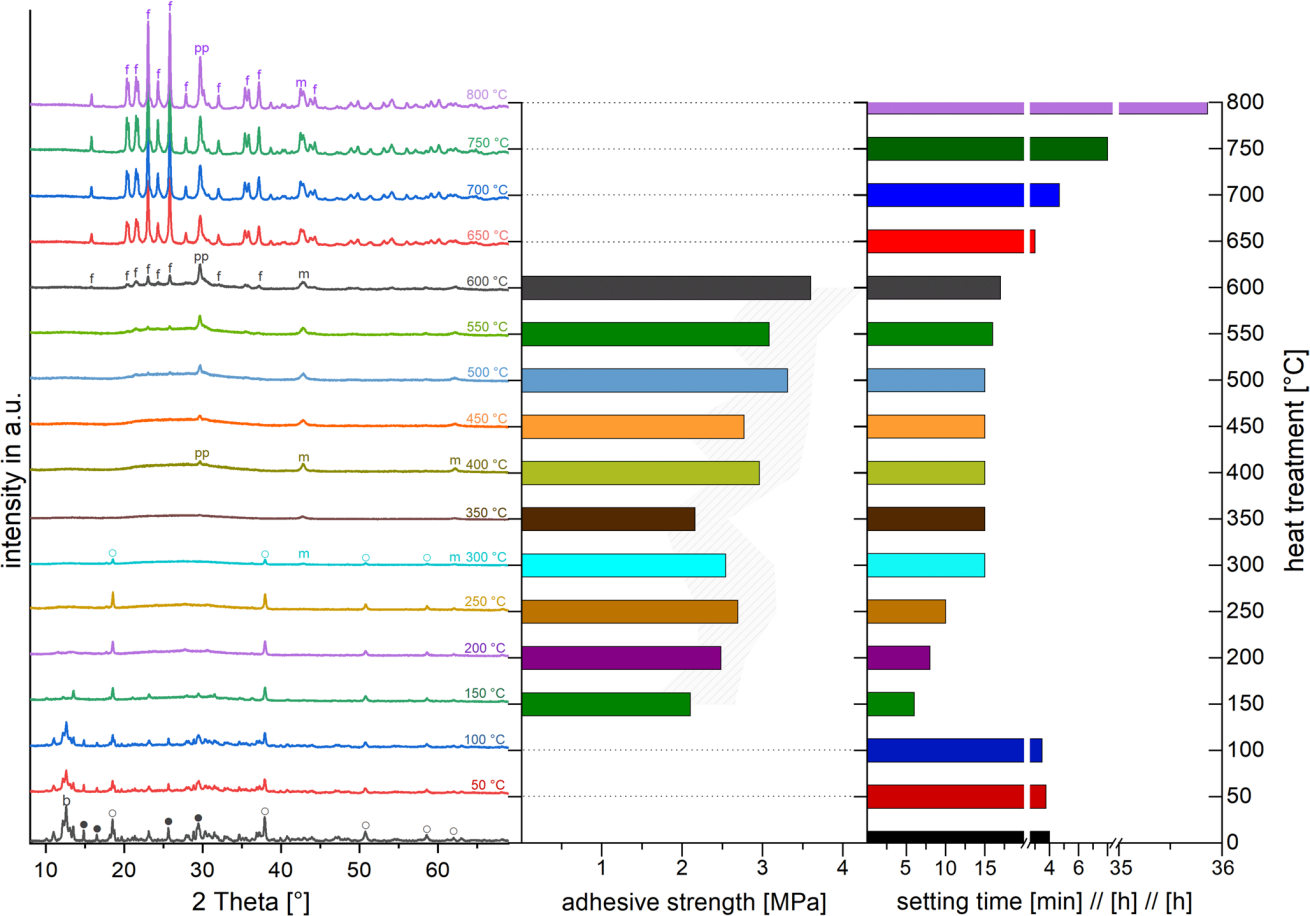
XRD patterns of Mg_3_(PO_4_)_2_∙ xH_2_O sintered at different temperatures (0-800 °C) as well as adhesive strengths (on jointed hydroxyapatite samples) and setting times when reacting with OPLS. f: Mg_3_(PO_4_)_2_ (farringtonite); pp: Mg_2_PO_2_O_7_; m: MgO; ◌: Mg(OH)_2_ (brucite); b: Mg_3_(PO_4_)_2_∙ 8H_2_O (bobierrite); ●: MgHPO_4_∙ 3H_2_O (newberyite)

Figure 3 illustrates the effects of the sintering temperature of the TMP hydrates (here TMP ∙ xH_2_O), in particular the associated changes in phase composition. XRD analysis revealed a multiphasic composition of the commercial TMP ∙ xH_2_O, such that no single defined hydration state can be assigned. Depending on the phase composition, Fig. 3 also shows the resulting changes in setting time and bond strength upon reaction with OPLS for use as a dental adhesive filling.

The unsintered precursor material TMP ∙ xH_2_O contains a mixture of phases, including magnesium hydrogen phosphate trihydrate (newberyite), magnesium phosphate octahydrate (bobbierite‑type), magnesium phosphate decahydrate (specific decahydrate phase), and magnesium hydroxide (brucite). The diffraction pattern of the Mg_3_(PO_4_)_2_ ∙ xH_2_O sintered at 100 °C largely corresponded to the crystalline starting material but showed additional peaks for bobierrite and newberyite. The setting time here was still more than 3 h, and no relevant adhesive strength could be meaningfully determined. From 150 °C, the setting time was significantly reduced to a measured minimum of only 6 min, with a general reduction in intensities and an increase in amorphous fractions. However, the adhesive strength at this temperature was the lowest recorded at 2.10 ± 0.55 MPa. Peaks for brucite occurred at 300 °C and 350 °C, and the setting time approached the 15 min considered optimal by the author. The adhesive strength of the sample sintered at 400 °C, which was used as a reference material, was measured at 2.96 ± 0.48 MPa, the highest among the compositions with a setting time of 15 min.

Above 400 °C, the material was predominantly amorphous with residual peaks for magnesium pyrophosphate and magnesium oxide. From 600 °C, peaks for farringtonite became visible, and the setting time remained below 20 min. At this temperature, the highest adhesive strength of 3.6 ± 0.65 MPa was recorded, although the desired viscosity decreased. From a sintering temperature of 650 °C, the phase fractions of farringtonite became more intense, and there was a sharp increase in the setting time to 3 h. A relevant adhesive strength could again not be meaningfully determined here. We chose Mg_3_(PO_4_)_2_ ∙ xH_2_O sintered at 400 °C for further investigations due to its optimal balance of setting time, adequate adhesive strength, and favorable handling properties.

A direct XRD comparison of TMP·xH₂O and cattiite before and after sintering at 400 °C is provided in the Supplementary Material (Fig. S2; [23]).

**Fig. 4:**
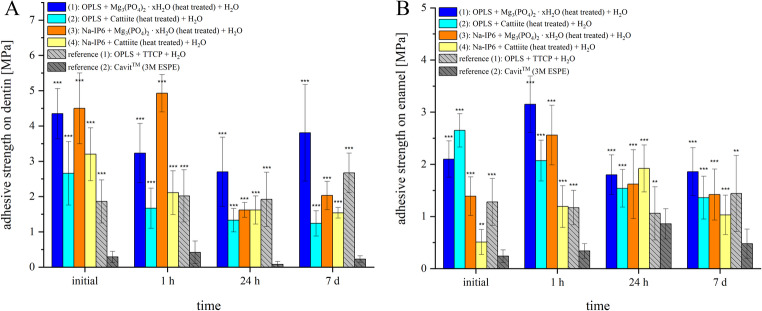
Shear strengths when bonding (**A**) dentin or (**B**) enamel with different formulations. blue: OPLS + Mg_3_(PO_4_)_2_ ∙ xH_2_O (heat-treated) + H_2_O. cyan: OPLS + cattiite (heat-treated) + H_2_O. orange: Na-IP6 + Mg_3_(PO_4_)_2_ ∙ xH_2_O (heat-treated) + H_2_O. yellow: Na-IP6 + cattiite (heat-treated) + H_2_O. Light gray (reference): OPLS + TTCP + H_2_O. dark gray (reference): Cavit™ (3M ESPE). Measurements were taken initially after the end of the setting time, after 1 h, after 24 h and after 7 d. Significance (Tukey HSD vs. Cavit™) is indicated by asterisks (**p < 0.01; ***p < 0.001). Other groupwise or time-dependent comparisons are omitted for clarity

After 7 days of storage, depending on the formulation, compressive strengths ranged from 5.2 MPa (lowest) to an average of 42.66 MPa (highest): (1) 14.76 ± 2.95 MPa; (2) 5.20 ± 0.96 MPa; (3) 42.66 ± 8.09 MPa; (4) 10.03 ± 3.47 MPa. The compressive strength of Cavit™ is classified in the literature as low at 13.8 MPa [24, 25]. The bond strengths of the cements tested are shown in Fig. 4. The materials containing TMP ∙ xH_2_O showed the highest shear strength when bonding dentin components, in particular the compound of TMP ∙ xH_2_O and Na-IP6 with 4.93 ± 0.53 MPa after 1 h, while Cavit™ exhibited the lowest values. However, the compound of TMP ∙ xH_2_O and OPLS with similar initial strength values shows relevantly higher shear strength values over time with 3.81 ± 1.37 MPa after 7 days of storage. The calcium phosphate-containing reference, consisting of pure-phase TTCP and OPLS (from patent No. US 8,273,803 B2 [26]; similarity to Tetranite^®^), showed an initial shear strength of 1.86 ± 0.61 MPa. Over the course of storage, this was increased to 2.67 ± 0.56 MPa. The comparatively very low adhesive strength of Cavit™, which did not exceed 0.48 ± 0.28 MPa after 7 days, is notable. These differences were confirmed statistically: one-way ANOVA for dentin revealed significant differences in bond strength between the materials (*p* < 0.001), with post-hoc Tukey analysis indicating that TMP-based adhesives (especially TMP ∙ xH_2_O/Na-IP6 and TMP ∙ xH_2_O/OPLS) consistently outperformed all other materials, while Cavit™ was significantly inferior to all other groups (*p* < 0.0001). The differences between the two TMP ∙ xH_2_O -based groups were minor and not consistently significant, whereas TTCP/OPLS performed intermediately—stronger than Cavit™, but weaker than TMP ∙ xH_2_O -based systems at most time points.

In general, the bone adhesives showed a comparatively lower strength on enamel. Adhesives containing OPLS initially showed slightly better performance with, for example, 2.65 ± 0.32 MPa for the combination of OPLS and cattiite. Initially, Cavit™ had the lowest strength at 0.51 ± 0.24 MPa. The highest value measured for the bonding of enamel was 3.15 ± 0.54 MPa for the compound of TMP ∙ xH_2_O and OPLS when measured after 1 h. A two-way ANOVA confirmed that material type, storage time, and their interaction significantly influenced enamel adhesion (all *p* < 0.0001). In post-hoc analysis, Cavit™ was significantly weaker than all other materials at every time point, while TMP ∙ xH_2_O/OPLS and cattiite/OPLS showed the highest values. Notably, cattiite/OPLS exhibited a significant time-dependent decline in shear strength (–1.28 MPa over 7 days, *p* < 0.001), while TMP/OPLS maintained high values over time. These findings highlight the relevance of both material composition and aging in determining adhesive performance on hard dental tissues. Detailed pairwise comparisons corresponding to Fig. 4 are provided in the Supplementary Material (Tables S1–S8).

The development of temperature and pH of the tested cements during setting is shown in Table 2. The temperature development during the setting process was similar for all four bone adhesives: Within the first 2–3 min, the setting temperature reached up to its maximum and then dropped rapidly again. Their maximum temperatures were between 31.4 °C and 37.2 °C. Figure 12 (appendix B.1) shows the pH dynamics and the temperature development of Cavit™ during setting. The maximum setting temperature was reached after 1–2 min and amounted to 26.6 °C. The initially measured pH value was 5.36, which increased continuously and approached asymptotically. After 24 h, the pH value was 6.66.

**Table 2:**
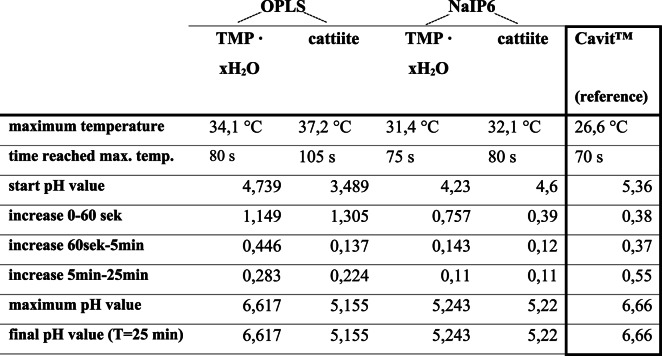
Summary of thermal and pH development parameters for different compositions

**Fig. 5:**
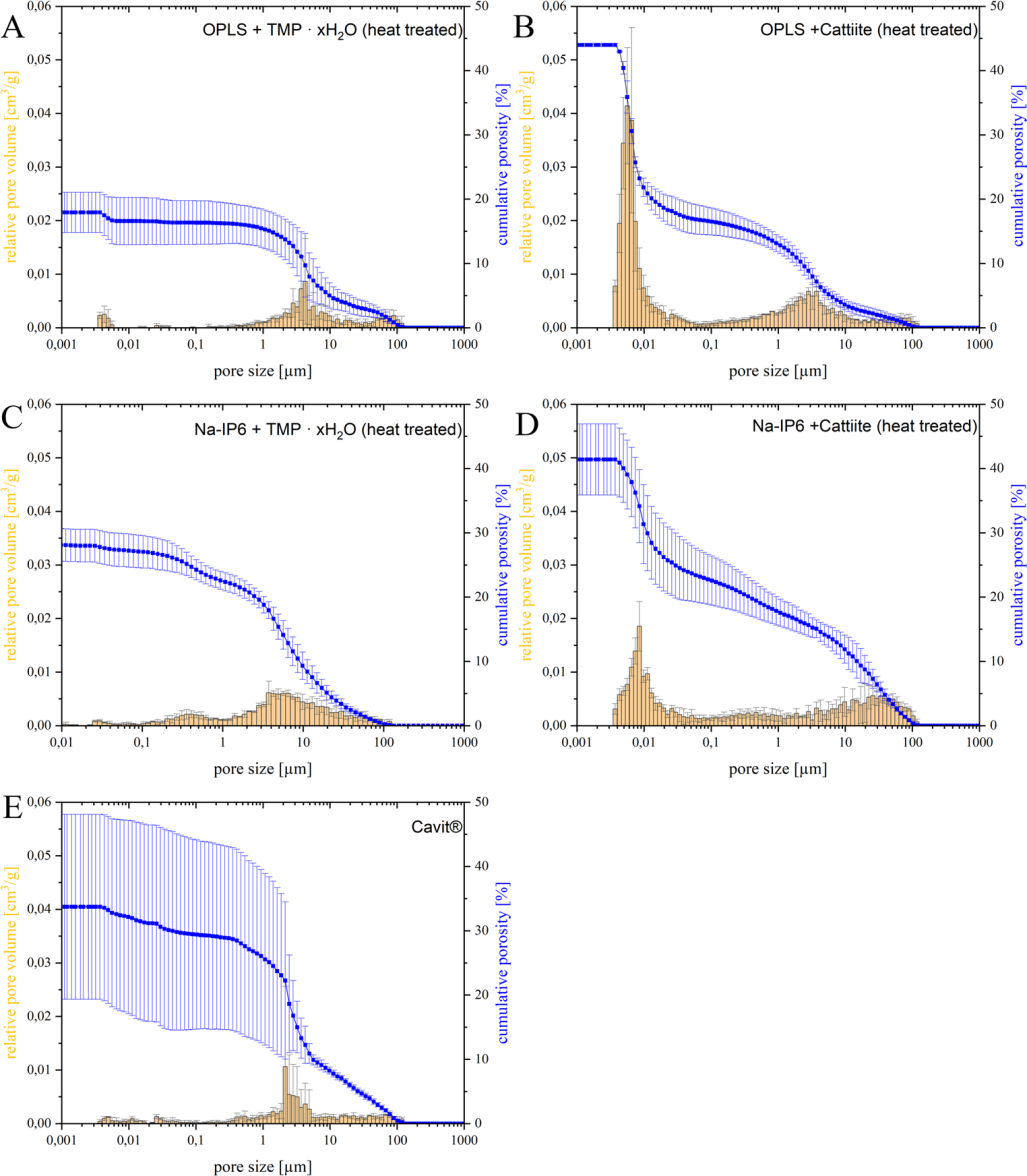
The cumulative porosity and the relative pore volume plotted against the distribution of the individual pore sizes. **A**: OPLS + TMP ∙ xH_2_O (heat-treated) + H_2_O. **B**: OPLS + cattiite (heat-treated) + H_2_O. **C**: Na-IP6 + cattiite (heat-treated) + H_2_O. **D**: Na-IP6 + TMP ∙ xH_2_O (heat-treated) + H_2_O. **E**: Cavit™ (3M ESPE)

The measurement results of the porosity measurement are shown in Fig. 5. At 17.9%, the compound of OPLS and TMP ∙ xH_2_O exhibited the lowest porosity of all tested samples with a multimodal pore size distribution (Fig. 5, A), while the counterpart with cattiite showed the highest porosity of 44% with a bimodal pore size distribution with maxima at 0.006 μm and 2.8 μm (Fig. 5, B). The reference Cavit™ (Fig. 5, E) achieved a porosity of 33.7%, almost twice as high as OPLS/TMP ∙ xH_2_O. Cavit™ showed a unimodal distribution with a maximum at 2.1 μm. All samples showed skewed distributions of pore sizes.

**Fig. 6:**
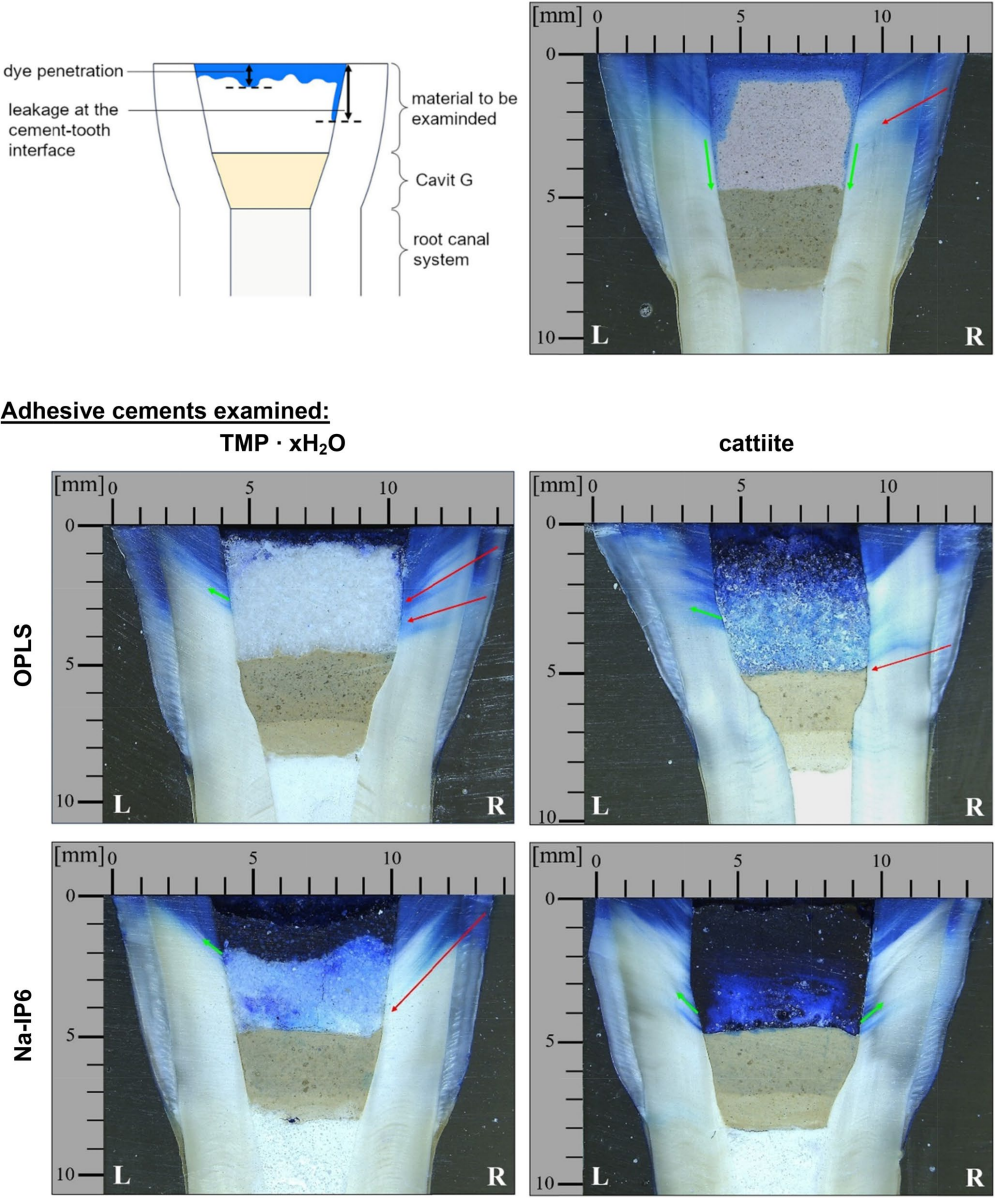
Microscopic images of the coronal part of the prepared root canal-treated bovine teeth stored in methylene blue. One representative sample is shown in each case (n=12). OPLS + TMP ∙ xH_2_O (heat-treated) + H_2_O; OPLS + cattiite (heat-treated) + H_2_O; Na-IP6 + TMP ∙ xH_2_O (heat-treated) + H_2_O; Na-IP6 + cattiite (heat-treated) + H_2_O; Reference: Cavit™ (3M ESPE). The diagram in the upper left corner shows how the infiltration of methylene blue at the cement-tooth interface and the bulk dye penetration were defined. Red arrow: Methylene blue penetrates from the outside via the dentinal tubules to the cement gap into the interior of the tooth. This is not to be interpreted as interfacial leakage. Green arrow: Methylene blue penetrates retrogradely via the cement gap into the dentinal tubules. This is a marker for the depth of the interfacial leakage

 Figure 12 (appendix B.2) shows overview images of the microsections and provides an overview of the fillers used, while Fig. 6 shows microscopic detail images. In the interpretation, a distinction was made between general dye penetration through the bulk material (hereafter referred to as ‘bulk dye penetration’), for example due to increased porosity of the filling material, and dye leakage at the interface between the filling material and the tooth. This differentiation can be difficult as the dye penetrates the dentin tubules. However, microscopic examination of these can differentiate whether the dye penetrates anterograde from the dentin surface in the direction of the pulp chamber or retrograde from the leakage at the filler/tooth interface. It is worth mentioning that such retrograde penetration can also be observed starting from the enamel-dentin junction, as in Fig. 6.

**Fig. 7:**
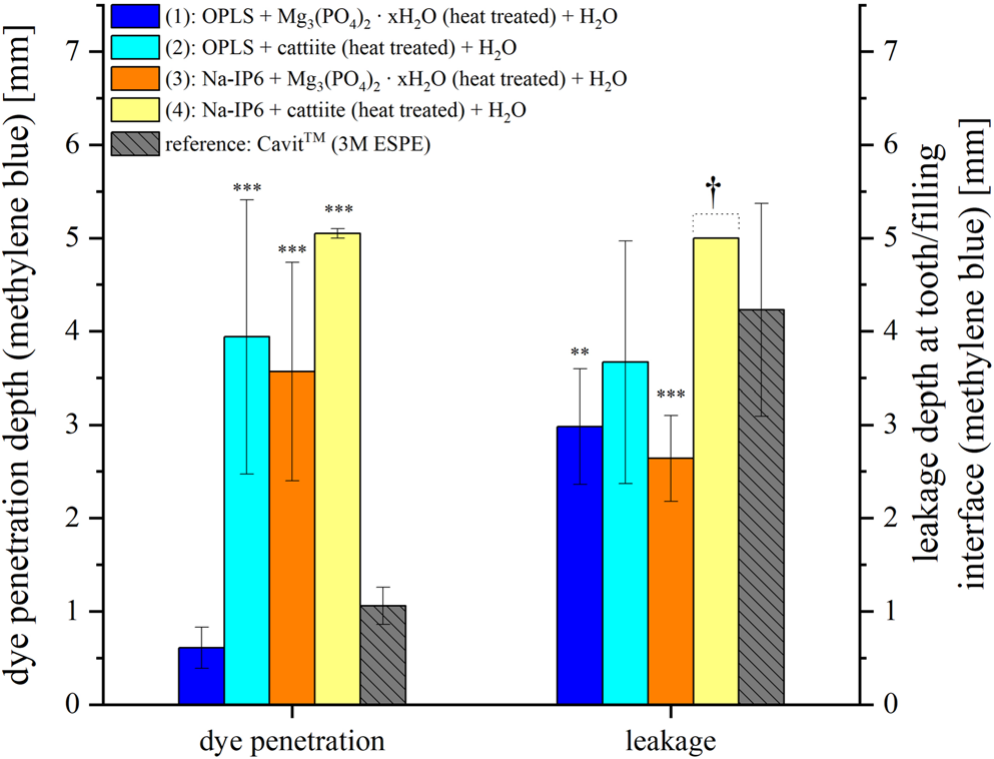
Measurement results of bulk dye penetration into the various covering fillings and leakage at the interface between tooth and covering filling. (1): OPLS + Mg_3_(PO_4_)_2_∙ xH_2_O (heat treated) + H_2_O. (2): OPLS + cattiite (heat treated) + H_2_O. (3): Na-IP6 + Mg_3_(PO_4_)_2_∙ xH_2_O (heat treated) + H_2_O. (4): Na-IP6 + cattiite (heat treated) + H_2_O. Reference: Cavit™ (3M ESPE).†: The interfacial leakage could not be assessed separately due to the high bulk dye penetration. Significance (Tukey HSD vs. Cavit™) is indicated by asterisks (**p < 0.01; ***p < 0.001). Other groupwise or time-dependent comparisons are omitted for clarity

 Figure 7 shows the quantitative evaluation of the methylene blue dye test. One-way ANOVA showed a highly significant effect of the material on dye penetration (p < 0.001). Leakage values also differed significantly between groups (p = 0.003). The lowest overall bulk dye penetration was observed with the compound of TMP ∙ xH_2_O and OPLS, while the compound of cattiite and Na-IP6 showed the highest penetration. In addition to the lowest bulk dye penetration of 0.61 ± 0.22 mm and a homogeneous appearance without cracks, the compound of TMP ∙ xH_2_O and OPLS showed a interfacial leakage of only 2.98 ± 0.62 mm. The compound of cattiite and OPLS as well as Cavit™ showed stronger bulk dye penetrations of 3.94 ± 1.47 mm and 1.06 ± 0.20 mm, respectively, with Cavit™ showing the highest interfacial leakage of 4.23 ± 1.14 mm. The compound of TMP ∙ xH_2_O and Na-IP6 showed a moderate bulk penetration of 3.57 ± 1.17 mm and a interfacial leakage of 2.64 ± 0.46 mm. The compound of cattiite and Na-IP6 showed the deepest bulk dye penetration of 5.05 ± 0.05 mm, whereby the interfacial leakage could not be measured precisely due to the intense coloration, but was at least 5 mm. Post-hoc analysis confirmed that bulk dye penetration was significantly lower for the TMP ∙ xH₂O and OPLS compound compared with most other materials, while not differing significantly from Cavit™, whereas TMP ∙ xH₂O -based systems exhibited significantly lower interfacial leakage than cattiite-based materials and Cavit™ (see Tables S9–S10 in the Supplementary Material for full pairwise comparisons). No dye penetrated into the root canal system in any of the samples, although there were clear differences in the resistance of the dye to penetration.

**Fig. 8:**
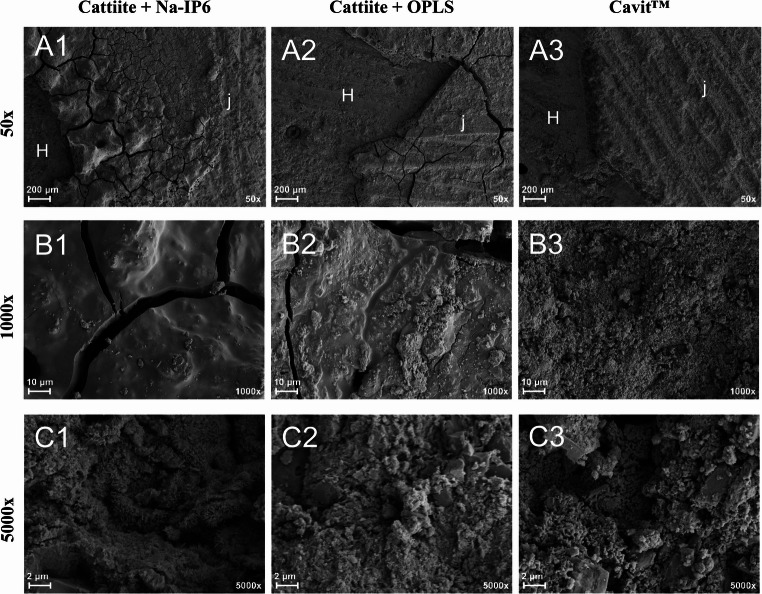
Scanning electron micrographs of adhesives that had bonded hydroxyapatite parts. Image were taken after failure in the testing machine. At 50x magnification, parts of both the adhesive joint (j) and the hydroxyapatite test specimen (H) are visible. Larger magnifications show only the adhesive. Left: Cattiite /Na-IP6 / H_2_O center: Cattiite /OPLS / H_2_O right: Cavit™ The hydroxyapatite test specimen was sanded with grit P 80. Images were taken at 50x (**A**), 1000x (**B**) and 5000x (**C**) magnification

 Figure 8 shows scanning electron micrographs of sheared, previously bonded parts. For the SEM images only, HA test specimens were bonded instead of teeth, as this is not permissible in the SEM at the institute due to the residual moisture. For reasons of simplicity, bonded joints containing TMP ∙ xH_2_O are not shown. They appeared similar to the congruents containing cattiite. SEM images of congruents containing TTCP have already been described elsewhere [15]. Visible cracks at the adhesive joint are due to the necessary intensive drying during sample preparation. Impressions of grinding facets of the formerly opposing adherends can be seen on all adhesive joints. This is an expression of good wetting and good flow properties and may indicate good sealing properties. Particularly at 5000x, clear crystal structures can be seen, predominantly occupying the upper right part of the image (in this case well visible in the compound of cattiite and Na-IP6; Fig. 8, C1).

**Fig. 9:**
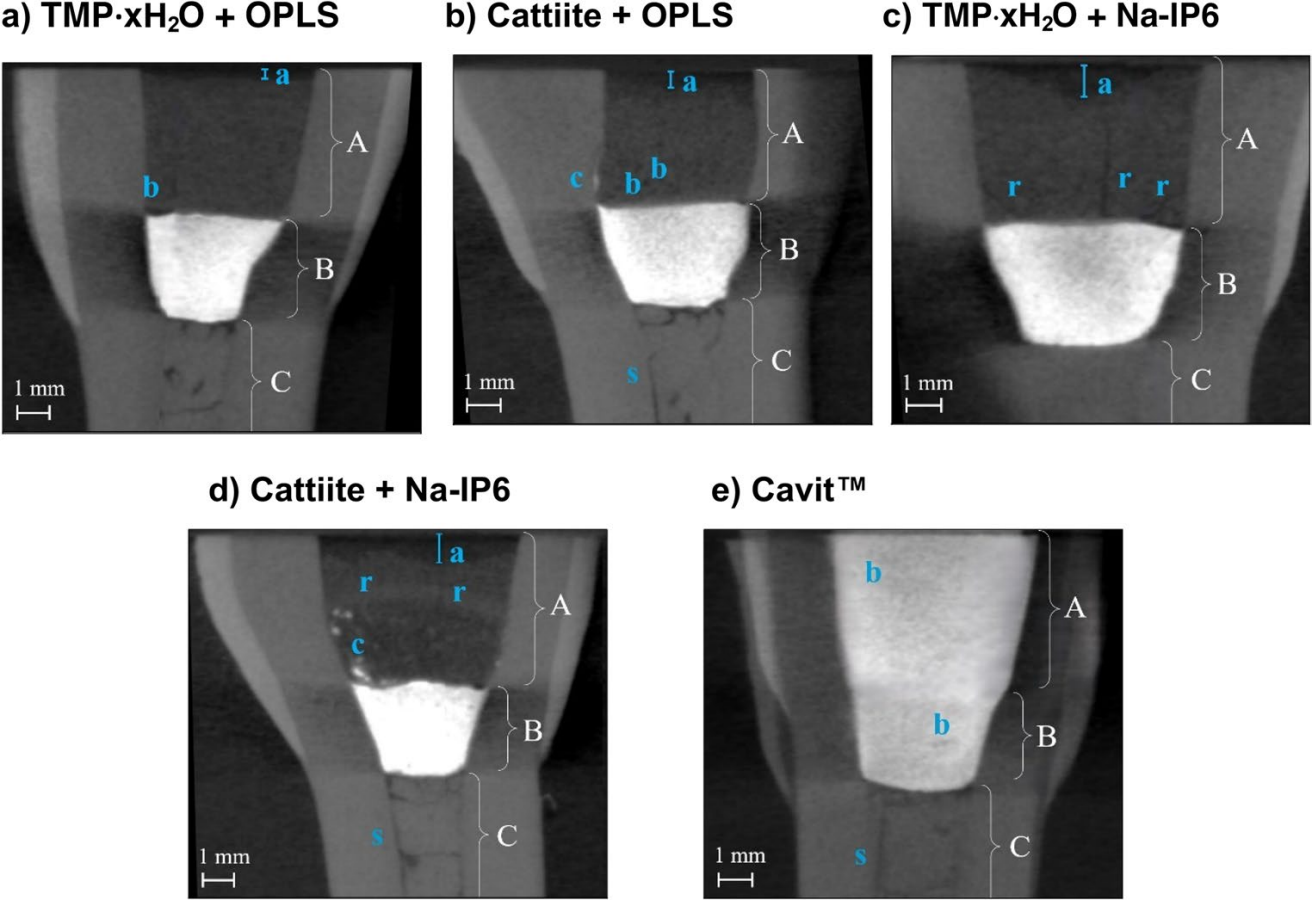
Micro-CT images of the different cover fillings, each in coronal view. (**a**): Mg_3_(PO_4_)_2_∙ xH_2_O (heat-treated) + OPLS + H_2_O. (**b**): Cattiite (heat-treated) + OPLS + H_2_O. (**c**): Mg_3_(PO_4_)_2_∙ xH_2_O (heat-treated) + Na-IP6 + H_2_O. (**d**): Cattiite (heat-treated) + Na-IP6 + H_2_O. (**e**): Cavit™ (3M ESPE). Designations: **A**: Magnesium phosphate cement, or Cavit™ for the reference test specimens; **B**: Cavit™ G; **C**: Ca(OH)_2_; **a**: Maximum leaching (**a**: 0.28 mm; **b**: 0.46 mm; **c**: 0.89 mm; **d**: 0.93 mm; **e**: 0.20 mm); **b**: Bubble; **c**: Cavit™ G residue; **s**: Gap; **r**: Crack

**Fig. 11:**
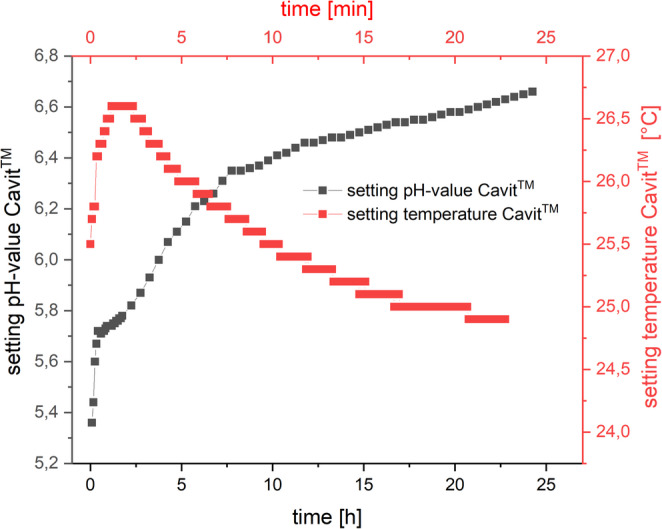
Setting temperature and pH value development during the setting of Cavit™ over time

**Fig. 12:**
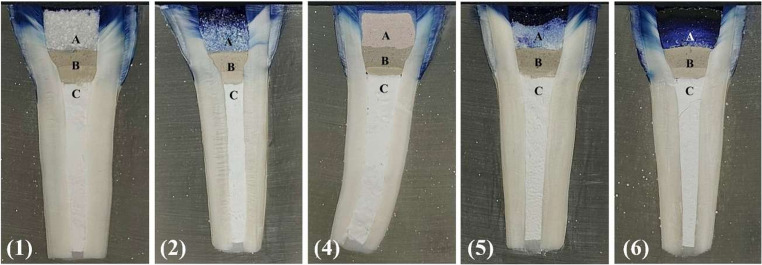
Microscopic overview images of the prepared root canal-treated bovine teeth stored in methylene blue. One representative sample is shown in each case (n=12). (1) OPLS + TMP ∙ xH_2_O (heat-treated) + H_2_O. (2) OPLS + cattiite (heat-treated) + H_2_O. (3) Cavit™ (3M ESPE). (4) Na-IP6 + TMP ∙ xH_2_O (heat-treated) + H_2_O. (5) Na-IP6 + cattiite (heat-treated) + H_2_O. **A**: Supposed bone adhesive, or Cavit™ ; **B**: Cavit™ G; **C**: Root canal system filled with Ca(OH)_2_

 Micro-CT examinations of endodontically treated anterior tooth samples were performed to supplement the analyses of barrier capacity and microleakage and to assess the intrinsic non-modified radiopacity. All tooth samples had undergone the above-mentioned 210 cycles of thermal cycling and liquid storage for 7 days on a platform shaker in methylene blue. Representative sections are shown in Fig. 9. No marginal gap formation between the covering filling and dentin was observed in any of the materials examined at a resolution of 30 µm. All magnesium phosphate-based compositions showed a lower radiopacity than dentin, while Cavit™ showed a comparatively high radiopacity. The combination of TMP ∙ xH_2_O and OPLS was characterized by a homogeneous cement structure, with a minimum washout of 0.3 mm. Congruent cements with cattiite showed inhomogeneities with bubbles and pores and a maximum washout of 0.5 mm. Cavit™ appeared relatively homogeneous with few air bubbles and hardly any washout. Combinations with Na-IP6 tended to crack. They also tended to have higher washouts, with a maximum measured of 0.9 mm in each case.

## Discussion

Commercially available TMP · xH₂O powder was heat-treated to enhance reactivity and control setting kinetics. Powders were thermally pretreated between 0–800 °C in 50 °C steps, and XRD was used to track phase evolution (see Fig. 3). The impact of the resulting mineralogical composition on cement properties was then correlated with setting time and shear strength data.

Below 100 °C, the powders contained mixed crystalline hydrates—primarily newberyite (MgHPO₄ · 3H₂O), bobierrite (Mg₃(PO₄)₂ · 8H₂O), and brucite (Mg(OH)₂). This is in agreement with thermodynamic predictions, where newberyite and bobierite exhibit low solubility and stability in near-neutral pH, particularly at ambient temperatures [27]. These phases showed poor reactivity toward phosphoserine, possibly due to limited ligand interaction or surface complexation capacity under cementation conditions, despite their moderate solubility.

While previous studies demonstrated rapid precipitation of crystalline newberyite from aqueous precursors under microwave irradiation [28], our data show that in dry, sintered powders newberyite is only detectable at low temperatures and disappears upon heating above 100 °C. At 150 °C, bobierrite also vanishes, leaving predominantly brucite phases. This transition marks the onset of significant dehydration, increasing surface reactivity and accelerating the setting reaction. Lothenbach et al. confirm that brucite is stable over a wide pH range but has limited hydraulic reactivity unless converted to MgO, which supports our findings [27]. At 250 °C, MgO began to form alongside brucite. MgO is considerably more reactive than brucite in aqueous environments, as it dissolves faster [29] and releases Mg²⁺ ions that can react efficiently with ligands such as phosphoserine, thereby supporting faster setting and improved cement formation.

 By 350 °C, brucite had decomposed, leaving mainly amorphous magnesium phosphate and MgO. This composition showed high surface reactivity, promoting Mg²⁺ release and interaction with phosphoserine, supporting rapid setting. The greater reactivity of amorphous magnesium phosphates has been reported previously [30], likely due to their disordered structure and higher density of reactive sites.

 At 400 °C, XRD additionally detected magnesium pyrophosphate, likely formed through dehydration and condensation reactions. A similar intermediate phase (MgH₂P₂O₇) has previously been observed in systems containing magnesium oxide and magnesium dihydrogen phosphate (Mg(H₂PO₄)₂), where it was shown to decompose to magnesium metaphosphate (Mg(PO₃)₂) between 250 and 550 °C [31]. Powders treated at this temperature achieved a favorable balance between setting time and shear strength (up to 3.6 MPa), with excellent handling and reproducibility. This favorable workability may be attributed to the presence of magnesium pyrophosphate, a poorly soluble and relatively unreactive phase that moderates the early setting reaction and thus facilitates easier and more controlled mixing.

 Above 550 °C, the dominant phase shifted to crystalline farringtonite (Mg₃(PO₄)₂), increasing in intensity up to 800 °C. Farringtonite is poorly reactive in its crystalline form and shows practically no setting in contact with water, prompting investigations into mechanical activation to enhance reactivity [32]. This has been attributed to its high crystallinity, strong phosphate binding, and low solubility. Lothenbach et al. report a very low solubility product (log Kₛₒ ≈ –22.4), confirming its thermodynamic inertness under standard cementation conditions.

 Once again, the 400 °C powder showed superior performance, combining rapid setting (~15 min), high shear strength (up to 3.6 MPa on hydroxyapatite joints), and excellent workability.For this reason, it was selected for mechanistic analysis of its reaction with phosphoserine, as outlined below. We propose the following reaction equation as a plausible reaction between TMP ∙ xH_2_O sintered at 400 °C and phosphoserine:

1. Thermal decomposition of TMP ∙ xH_2_O:
  1 $$\begin{array}{l}2Mg_3{(PO_4)}_2\cdot xH_2O\;\xrightarrow{400^\circ}C\;Mg_3{(PO_4)}_2^{(amorphous)}\\+\;Mg_2P_2O_7\;+\;MgO\;+\;xH_2O\uparrow\end{array}$$
2. Release of magnesium ions in water:
  2.1 $$MgO\;+\;H_2O\;\;Mg^{2+}\;+\;2OH^-$$
  2.2 $$Mg_3{(PO_4)}_2^{{{(amorphous})}}\;+\;2H_2O\;\rightleftharpoons\;3Mg^2+\;+\;2HPO_4^{3-}\;+\;2OH^-$$
  2.3.1 $$Mg_2P_2O_7\;(s)\;\rightleftharpoons\;2Mg^{2+}\;(aq)+\;P_2O_7^{4-}\;(aq)P_2O_7^{4-}\;+\;H_2O\;\rightleftharpoons\;2\;HPO_4^{2-}$$
  2.3.2 $$P_2O_7^{4-}\;+\;H_2O\;\rightleftharpoons\;2\;HPO_4^{2-}$$
3. Complex formation with phosphoserine:
  3 $$nMg^{2+}\;+\;mC_3H_8NO_6P\rightarrow\;\lbrack Mg_n{(C_3H_8NO_6P)}_m\rbrack$$

Alongside the previously described adhesion tests on hydroxyapatite, this study also includes tests on dental hard tissue. To the best of our knowledge, this study is the first to evaluate declared bone adhesives in in application on dental hard tissue. While no test standard exists for the discipline of bone adhesives, standards for determining adhesion are established in the field of dentistry. “Dentistry - Adhesion - Notched-edge shear bond strength test” (ISO 29022:2013) [33] is an international standard that describes a shear test method. It can be used to determine the shear bond strength between dental restorative materials and enamel or dentin. Nevertheless, the process described mainly involves the use of adhesive layers, such as when using composite materials. Also according to the description of the test standard, the procedure can only be used with other adhesive restorative materials such as glass ionomer cements with appropriate modification. Corresponding preliminary tests for this study confirmed this statement, as the required bonding surface is too small to be practicable for cementitious materials. In order to achieve comparability of the materials with regard to their adhesion to bone, the test specimen geometry and test procedure were based on previous work on bone adhesives [15, 22]. This test method at least conforms to many aspects of ISO 29022:2013 and is to be interpreted as a corresponding modification.

 The results of the shear strength tests clearly highlight the main motivation behind this study: in comparison to Cavit™, the tested bone adhesives exhibited significantly higher adhesive strength, as Cavit™ shows virtually no measurable adhesion. According to the authors, adhesive cements offer particular advantages in conical, non-retentive cavities, where they are presumably less prone to dislodgement. This could also enable a simplified clinical workflow, allowing non-solid spacers such as foam pellets to be placed without compromising retention—since the adhesive properties of the cement may help prevent loss of the provisional under masticatory load. When comparing the adhesive cements to one another, it becomes evident that formulations containing cattiite show lower bond strengths than their TMP ∙ xH₂O based counterparts. Cattiite was intentionally included as a structurally well-defined reference phase to enable mechanistic comparison. The results emphasize that chemical similarity alone is insufficient to predict performance, and that phase composition, porosity, and thermal transformation products critically govern cement reactivity and adhesion. In this context, the inferior performance of cattiite-based formulations may be attributed to the fact that, after sintering at 400 °C, cattiite is fully amorphous, whereas TMP ∙ xH₂O retains a partially crystalline structure—an aspect that, as previously discussed, may promote reactivity and enhance adhesion. The reference cement composed of OPLS and TTCP represents a common type of mineral–organic calcium phosphate-based adhesive described in the literature. In this study, however, the OPLS and TMP ∙ xH₂O formulation showed significantly higher shear strength at all time points. This is likely due to the higher reactivity of the heat-treated TMP, its favorable setting pH close to physiological conditions, and the formation of strong chelate complexes between Mg²⁺ ions and the functional groups of phosphoserine, which promote cohesive failure modes and enhance adhesion, as discussed in detail in our previous study [15]. Na-IP6 based adhesive cements exhibited significantly higher shear strengths at early time points compared to OPLS based formulations. However, this adhesive strength decreased markedly over time. The underlying causes for this behavior remain speculative. Micro-CT images revealed pronounced leaching in both Na-IP6 groups, with maximum bulk dye penetration depths of 0.89 mm and 0.93 mm (see Fig. 9), which may indicate increased hydrolytic sensitivity. Additionally, cements showing declining shear strength also exhibited more frequent crack formation, potentially caused by volumetric changes such as shrinkage, which could compromise adhesion.

 The investigated adhesive cements reached maximum temperatures between 32.1 °C and 37.2 °C at room temperature within 75–105 s, exceeding that of the reference material Cavit™ (26.6 °C after 70 s). This temperature increase can be considered low to moderate and is clinically irrelevant in the endodontic context due to the absence of pulpal tissue. Even in cases involving vital pulp, a harmful rise in temperature appears unlikely, as the low thermal conductivity of dentin may insulate deeper tissues from thermal insult. However, for potential applications such as direct or indirect pulp capping, the exothermic reaction should be taken into account. Zach and Cohen [34], a frequently cited and foundational study, reported that an intrapulpal temperature increase of 5.5 °C may lead to irreversible pulpitis and should therefore be avoided, as it could raise pulpal temperatures above the critical threshold of 42.4 °C. However, in vitro studies likely overestimate thermal stress on the pulp, as they lack physiological factors such as pulpal blood flow, dentinal fluid movement, and surrounding periodontal tissues [35–37]. Temperature elevations above 43 °C may activate pulpal nerve fibers, inducing a reactive increase in blood flow that supports heat dissipation [38]. Still, the overall contribution of pulpal perfusion to thermal regulation is considered limited due to the relatively low intrapulpal blood volume [38]. In addition to thermal effects, the pH of the materials may also influence their clinical applicability. After 25 minutes, the tested cements reached pH values between 5.2 and 6.7. For their intended use as temporary fillings in endodontic treatments, these values are unlikely to affect dental hard tissue, similar to Cavit™, which has a comparable pH. However, again in a hypothetical application as direct pulp capping materials, the initially lower pH could be of relevance. MTA is frequently used for this purpose. However, in contrast to the adhesive cements presented here, it exhibits a strongly alkaline pH of approximately 12 [39] —representing a far greater deviation from physiological pH than the moderately acidic to near-neutral values observed in our materials—and maintains this high pH over an extended period (at least 24 hours [39]). Its clinical success might therefore attributed not only to pH but also to its bioactivity and regenerative properties. Likewise, the more moderate pH of the tested cements—combined with rapid setting and potential biocompatibility—may still support pulpal healing in future applications.

 Looking at the porosimetry results (see Fig. 5), it is noticeable that the combination of OPLS and TMP ∙ xH₂O has the lowest porosity. This combination may result in lower porosity due to the high reactivity of the heat treated magnesium phosphate hydrate, efficient network formation by OPLS, and a relatively low water demand (PLR =3,53). In contrast, the strong hydrophilicity and chelating properties of Na-IP6 likely increase fluid requirements and disrupt cohesive network formation, promoting pore development.

 In the present study, an in vitro test of sealability using methylene blue was chosen. The in vitro testing of the sealability of endodontic temporary fillings was criticized already in 1993 due to inconsistent results [40]. From a purely ethical point of view, however, such tests are essential before being carried out in vivo. Anyhow, they can be used primarily for comparative purposes within the methodology of a study. Under “Wanted: A Base of Evidence”, the Editorial Office of the Journal of Endodontics even proposed a moratorium on sealant studies directly comparing endodontic techniques in 2007 [41]. For example, publications that do not demonstrate the clinical implications in order to promote the establishment of a gold standard are also rejected [41]. Such a standard method has not yet been established and the methodology behind it, together with its clinical relevance, must therefore be discussed in each case. The use of dye with sample immersion is the most commonly used method [42]. Other methods include the use of bacteria, glucose, radioisotopes or electrochemical methods, while a dual chamber apparatus is also sometimes used as a test technique. Considering pathogenic microorganisms, their by-products and nutrients, tracers such as methylene blue or glucose, which take all these factors into account, are preferred [42]. The required similarity of the test setup to clinical scenarios is discussed below. The outer surface of the tooth samples was sealed with a coating as demanded [42]. At the time of this study, neither a preferred material nor a standardized technique for temporary closure could be identified in the literature research, which complicates the comparability of the studies. Cavit™ is widely used and recommended by clinicians for a minimum thickness of 4 mm [43]. Combinations of different cover materials in the sense of a “double seal” technique are also widespread [44]. Wuersching et al. (2023), for example, attested to the combination of Cavit™ *white* with a composite as having superior sealing properties [45]. From the authors’ point of view, however, the use of composites in this context is sometimes prohibitive, as practitioners often prefer uncomplicated, easily removable and economical solutions. Here, a double layer technique has been chosen as a plausible clinical scenario. While Cavit™ G is an inexpensive, easy-to-use, easily removable material that forms a tight barrier close to the root canal, an adhesive cement is placed on top, which is just as easy to remove by the dentist, but can also prevent filling loss in conical cavities due to its adhesive properties. The use of layers of a defined size also ensures greater control, valid comparability and functional separation of properties. The decision to use methylene blue was made for the reasons mentioned above and for 1 week after the previous 210 cycles of thermocycling. As just one of numerous examples, Imura et al. showed in an in-vitro study that neither the use of gutta-percha, IRM or Cavit was able to prevent the penetration of microorganisms from the saliva into obturated canals over a test period of 22 days [46]. It is commonly known that the sealing quality of available temporary endodontic materials decreases with time and it seems advisable in clinical practice not to plan more than 1 week between visits for Cavit™ fillings [47]. The number of cycles within thermocycling is difficult to correlate with the retention time of restorations in the mouth, but an estimate of around 10,000 cycles per year or 20-50 cycles per day is suggested [48]. The suggested number of cycles to be assigned to a time period varies greatly in the literature and often significantly fewer cycles are proposed [49]. The temperature range used of 5°C-55 °C corresponds to the values normally used [49]. In summary, the methodology used, such as the configuration of the cover fillings, the thermocycling of 210 cycles (defined as at least 1 week of aging in the mouth) and the subsequent storage in methylene blue on a platform rotator for 1 week, conforms to the clinical practice and scientific guidelines that could be identified. Looking at the results of the microleakage test (see Figs. 6 and 7) and the shear strength (see Fig. 4), no correlation between adhesion and microleakage can be identified. A possible explanation for the lack of correlation lies in material-specific properties such as shrinkage, expansion, and porosity. Cavit™, for example, undergoes hygroscopic expansion, which may enhance marginal sealing [50, 51]. This expansion could theoretically induce internal stress and has been discussed as a potential factor in crack formation, although direct evidence for such an effect in Cavit™ is lacking. However, other materially related hygroscopically expanding materials such as Coltosol® F have been associated with a formation of tooth fractures in vitro [52]. In addition, increased porosity in the material can allow the tracer to penetrate independently of the adhesive force, which is clearly demonstrated once again by our own observations mentioned above (see Fig. 5 and Fig. 6). In this context, it is important to distinguish between bulk dye penetration and interfacial microleakage. While dye penetration may occur through the material itself via intrinsic porosity or microcracks, microleakage refers specifically to tracer penetration along the tooth-material interface and does not necessarily correlate with bulk penetration. Irrespective of the inherent porosity of a material, microcracks can also form in highly adhesive materials, which may shrink under thermocyclic conditions and compromise the marginal integrity. Independent of a material’s inherent porosity, microcracks may form in highly adhesive cements due to shrinkage during thermocycling, potentially compromising marginal integrity. Micro-CT analysis revealed the presence of such microcracks predominantly in adhesive cements that exhibited inferior sealing ability in the dye leakage tests (see Fig. 9). All these observations emphasize that evaluating sealability requires a comprehensive assessment of both mechanical properties, such as adhesion, and structural characteristics, including porosity and crack formation. Achieving reliable sealability depends on finding the right balance between volumetric stability and slight expansion—enough to maintain marginal integrity, but not so much as to risk crack formation in the surrounding tooth structure.

 The sodium phytate-containing adhesive cements show a particularly high leakage (see Fig. 6 and Fig. 7), which correlates with the formation of cracks and increased washout in micro-CT. Among the OPLS-based adhesive cements, it is noteworthy that the combination of TMP ∙ xH_2_O and OPLS exhibits significantly lower microleakage compared to the combination of cattiite and OPLS, which is initially surprising given the chemical similarity of these magnesium phosphates. A likely explanation lies in the porosity, which is significantly higher when using cattiite (see Fig. 5). This is reflected not only visually (see Fig. 6, “cattiite / OPLS”) but also in the quantitative analysis (see Fig. 7). The absence of crack formation, presumably due to lower shrinkage, as well as the reduced porosity, which limits bulk dye penetration within the material, seem to be the reasons why the combination of OPLS and heat-treated TMP ∙ xH_2_O performs well in terms of bulk dye penetration and interfacial leakage. This performance is comparable to Cavit™ and, under the limitations of the sample set and study design, may even slightly exceed it. At this point, it is important to consider the color intensity of the dye, as shown by Wu et al. (1998), who demonstrated that methylene blue loses intensity when in contact with materials such as zinc oxide-eugenol cements (Cavit™) or hydroxyapatite [53]. This effect is clearly visible in the present study, especially when comparing the different color intensities of the methylene blue, for example at the coronal part of the fillings or at the cement-tooth interface (see Fig. 6). This factor should be taken into account when evaluating the results. As previously outlined, a direct comparison of microleakage outcomes from dye penetration tests across different studies is not permissible due to methodological heterogeneity. According to Abdin and Al-Tayyan [54], contradictory results of in vitro leakage studies may not be due to their unreliability, but rather to differences in methodological variables and clinically relevant variables between them. In this context, comparing the applied methodology with related investigations remains meaningful in order to contextualize the present findings within the existing body of research.

 To the authors opinion, several key methodological parameters must be considered when assessing dye penetration with methylene blue. A major factor is dye concentration, which typically ranges from 0.5% to 2%—e.g., 0.5% in Adnan and Khan [55], 1% in Devi and Khan [56], and 2% in Çiftçi et al. [57] and Thu et al. [58]—with rare outliers such as 10% in Teplitsky et al. [59] Storage duration and conditions also vary considerably, from 10 minutes [55] to 7 days [57, 59] or 10 days [58], with all aforementioned studies using static storage. Thermocycling protocols differ as well, e.g. 150 cycles in Adnan and Khan [55], 500 cycles in Çiftçi et al. [57] or no thermocycling in Thu et al. [58] or Devi and Khan [56]. Such methodological differences must be considered when comparing outcomes. This study employed 2% methylene blue, 260 thermocycles, and 7-day storage, which overall falls within the range of parameters commonly reported in the literature. Unlike previous studies, the specimens were stored under continuous agitation on a platform shaker, which the authors consider more representative of clinical conditions. The authors consider the applied testing conditions to be relatively stringent. As outlined above, direct comparison of dye penetration studies must be interpreted with caution. Nevertheless, the study by Çiftçi et al. [57] offers a useful point of reference due to several methodological similarities, including the use of 2% methylene blue, 7-day storage, thermocycling, and a 4 mm filling depth. Key differences include the use of human premolars in Çiftçi et al., and the use of a cotton pellet instead of a bottom filling. Consistent with our results, Çiftçi et al. found that no material fully prevented dye penetration. Cavit™ G showed leakage into the material and along the cavity walls, in isolated cases reaching the base of the filling. This agrees with our observations for Cavit™, though in some cases we also noted penetration into the bottom filling. Our best-performing cement (TMP ∙ xH_2_O + OPLS) showed similar interface behavior but no dye reached the substructure in any specimen, indicating a better seal. Furthermore, Çiftçi et al. reported full dye penetration into the cotton pellet with IRM (a polymer-reinforced zinc oxide-eugenol material) and Ketac™ Molar Easymix (a dual-curing glass ionomer cement). In our study, only the weakest composition (Na-IP6 + Cattiit) showed comparably extensive leakage.

 This study is subject to certain limitations. All experiments were conducted in vitro and therefore cannot fully reproduce the complex biological and mechanical conditions encountered in vivo. In addition, microleakage testing remains method dependent and primarily allows comparative interpretation within a given experimental setup. Although thermocycling was applied as an aging protocol, longer-term aging as well as combined mechanical and biological influences were not addressed. While shear bond strength testing enabled standardized assessment of interfacial adhesion, future studies should further investigate clinically relevant dislodgement mechanisms using push-out or pull-out testing in standardized access-cavity geometries and include extended aging or in vivo evaluation.

## Conclusion

 This study introduced a novel class of mineral–organic adhesive cements composed of magnesium phosphate hydrates and organophosphates, originally developed for bone applications, and evaluated their potential use as temporary endodontic filling materials. Among all tested formulations, the most favorable performance was achieved by combining O-phospho-L-serine (OPLS) with trimagnesium phosphate hydrate (TMP · xH₂O) that had been thermally treated at 400 °C.

 X-ray diffraction revealed that this specific heat treatment yielded a multiphase composition containing amorphous magnesium phosphate, magnesium oxide (MgO), and magnesium pyrophosphate (Mg₂P₂O₇), which together provided high reactivity and a balanced setting profile. In combination with OPLS, this formulation showed excellent handling, a clinically acceptable setting time (~15 minutes), and the highest compressive strength (42.7 ± 8.1 MPa). In shear bond strength testing, it significantly outperformed the clinical gold standard Cavit™, achieving 4.35 ± 0.71 MPa on dentin and 2.10 ± 0.35 MPa on enamel shortly after setting, compared to Cavit™ (0.29 ± 0.16 MPa on dentin; 0.24 ± 0.12 MPa on enamel). Sealing performance was comparable to or better than Cavit™, considering the general limitations of methylene blue dye penetration tests.

 These findings suggest that magnesium–phosphoserine cements, particularly the formulation based on 400 °C-treated TMP · xH₂O, are promising candidates for use in non-retentive cavities, where conventional materials often fail. Their adhesive properties, removability, and ease of handling also support broader potential applications in dentistry, such as pulp capping or perforation repair. This study provides a strong foundation for future preclinical and clinical investigations.

## Supplementary Information

Below is the link to the electronic supplementary material.

ESM 1 (DOCX 254 KB)

## Figures and Tables

**Fig. 10:**
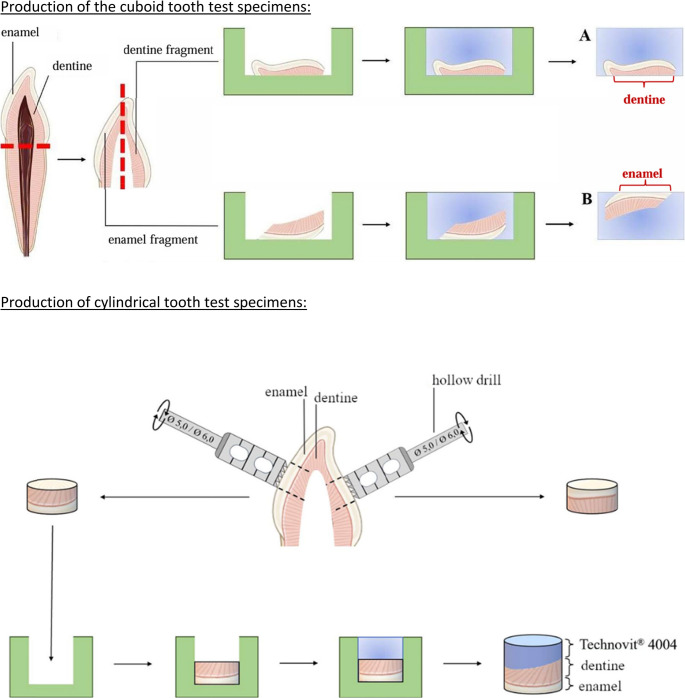
Preparation of the respective cuboid and cylindrical tooth fragments and illustration of the subsequent embedding procedure to create dentin test specimens (**A**) and enamel test specimens (**B**) for testing the bond strength

**Fig. 13:**
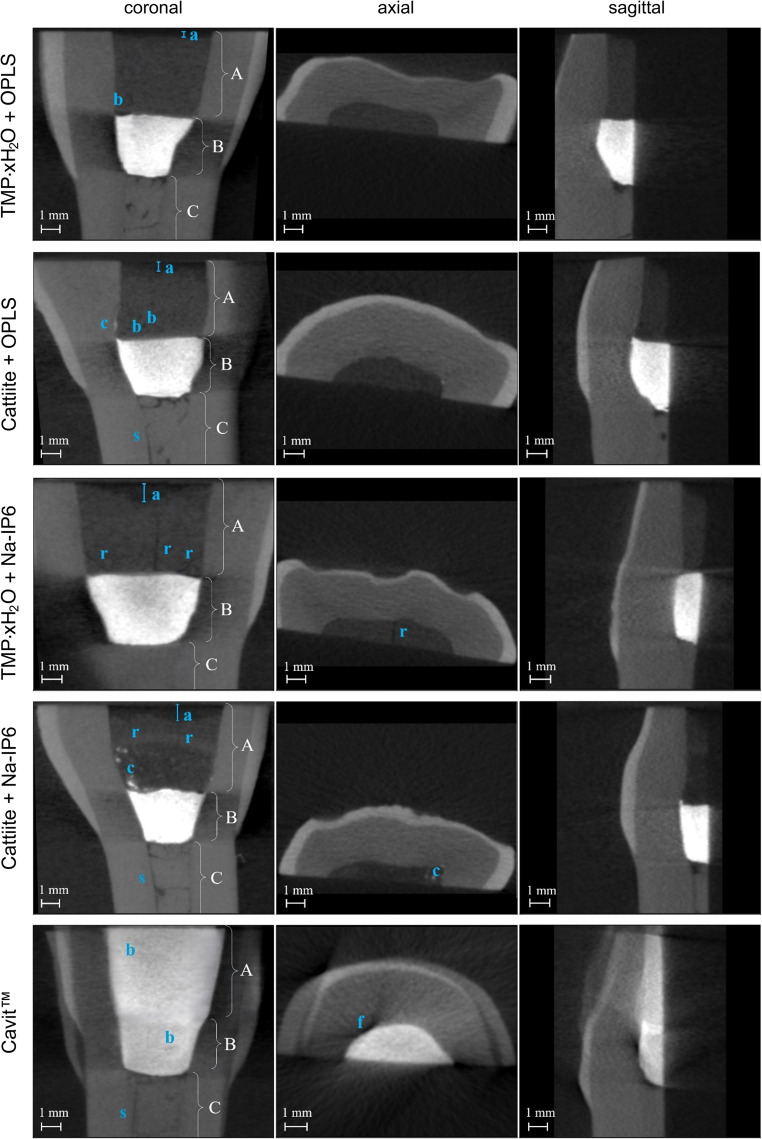
Micro-CT images of the different cover fillings, each in coronal (left), axial (center) and sagittal (right) view. (1): Mg_3_(PO_4_)_2_∙ xH_2_O (heat-treated) + OPLS + H_2_O. (2): Cattiite (heat-treated) + OPLS + H_2_O. (3): Mg_3_(PO_4_)_2_∙ xH_2_O (heat-treated) + Na-IP6 + H_2_O. (4): Cattiite (heat-treated) + Na-IP6 + H_2_O. (5): Cavit™ (3M ESPE). Designations: **A**: Magnesium phosphate cement, or Cavit™ for the reference test specimens; **B**: Cavit™ G; **C**: Ca(OH)_2_. **a**: depth of leaching; **b**: bubble; **c**: Cavit™ G residue; **s**: Gap; **r**: Crack; **f**: artifact

## Data Availability

The data supporting this study are available from the corresponding author upon reasonable request.
