# Supplementary material for: Magnesium-organophosphate bone adhesives repurposed as endodontic cements for dental applications
Source: Clin Oral Investig. 2026 Feb 4;30(2):70. doi: 10.1007/s00784-026-06743-9 (PMC12872749; doi:10.1007/s00784-026-06743-9)
Supplement: Supplementary file 1 — ESM 1 (DOCX 254 KB) [file 784_2026_6743_MOESM1_ESM.docx]

Supplementary Material

| 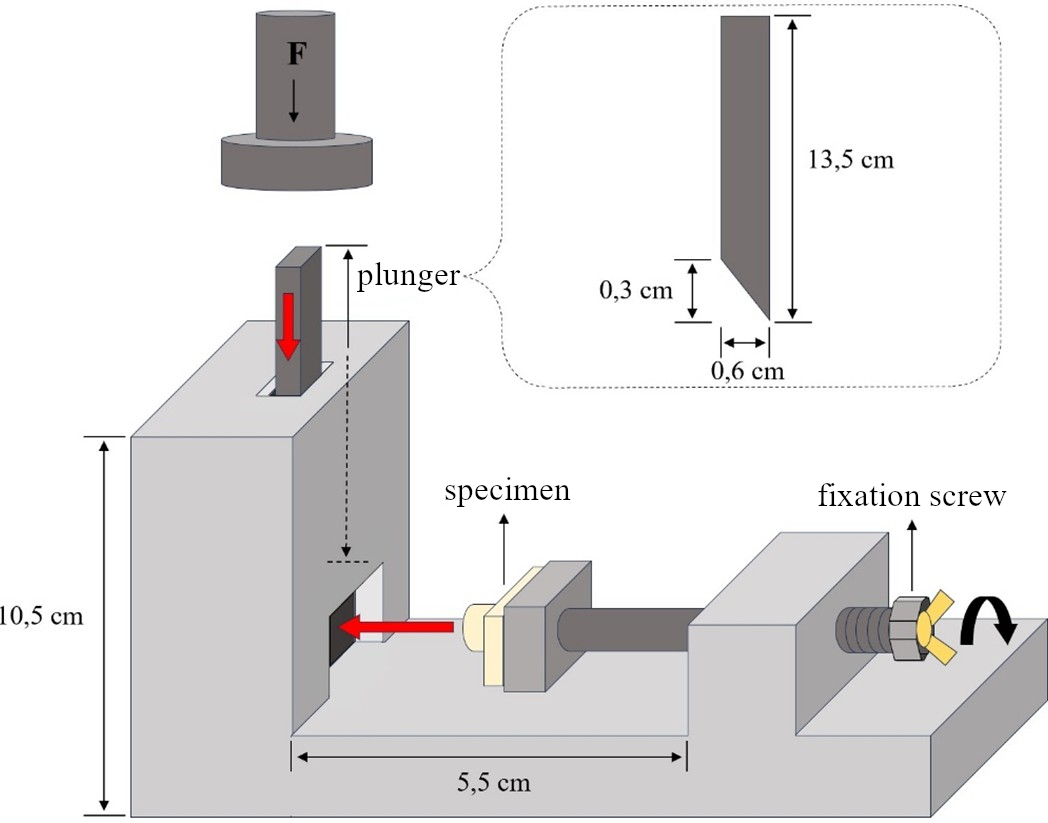 |
| --- |
| Figure S1: Schematic illustration of the custom-built shear testing device used for determination of shear bond strength, based on a device previously described in [15,22]. A loading plunger applies a controlled lateral force to the test specimen, which consists of two bonded parts and is secured in position by a fixation screw. |

| 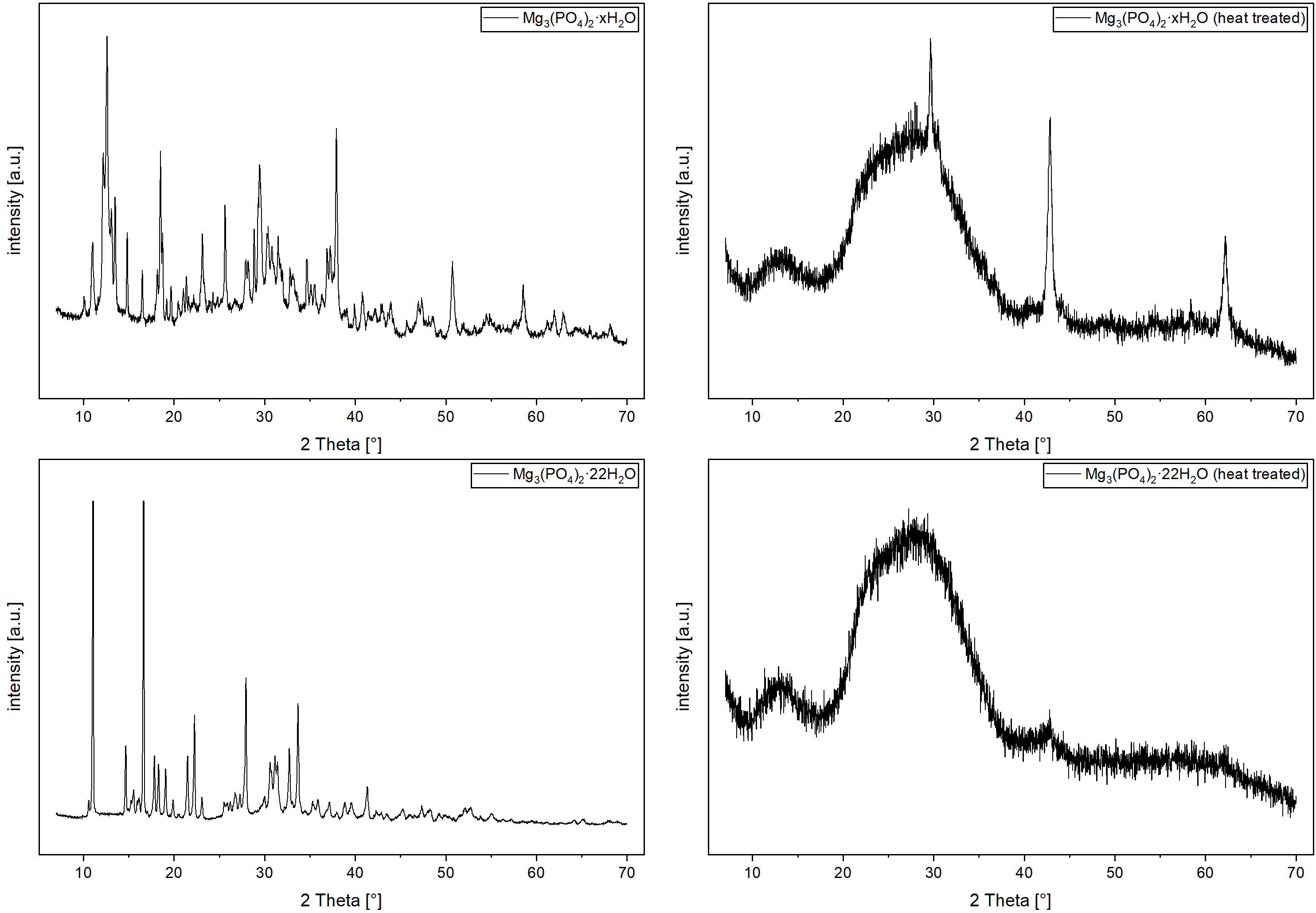  A  D  B  C |
| --- |
| Figure S2: x-ray diffraction patterns of multiphase Mg₃(PO₄)₂·xH₂O and Mg₃(PO₄)₂·22H₂O (cattiite) before and after heat treatment. (A) As-received commercial Mg₃(PO₄)₂·xH₂O, (B) Mg₃(PO₄)₂·xH₂O after sintering at 400 °C, (C) precipitated cattiite (Mg₃(PO₄)₂·22H₂O) prior to thermal treatment, and (D) cattiite after sintering at 400 °C. The diffraction data were reported previously in [23], but are re-plotted here from the original raw data to enable direct comparison in the context of the present dental application. |

Table S 1: Post-hoc pairwise comparisons of initial shear strength values on dentin between the different material formulations. Adjusted p-values (Tukey HSD) and corresponding significance levels are reported. n.s., not significant; *p < 0.05; **p < 0.01; ***p < 0.001; n = 12 per group.

| Comparison | p (adj.) | Significance |
| --- | --- | --- |
| TMP∙xH_2_O/Na-IP6 vs TMP∙xH_2_O/OPLS | n.s. | – |
| TMP∙xH_2_O/Na-IP6 vs Cattiite/Na-IP6 | <0.001 | *** |
| TMP∙xH_2_O/Na-IP6 vs Cattiite/OPLS | <0.001 | *** |
| TMP∙xH_2_O/Na-IP6 vs TTCP/OPLS | <0.001 | *** |
| TMP∙xH_2_O/Na-IP6 vs Cavit™ | <0.001 | *** |
| TMP∙xH_2_O/OPLS vs Cattiite/Na-IP6 | <0.001 | *** |
| TMP∙xH_2_O/OPLS vs Cattiite/OPLS | <0.001 | *** |
| TMP∙xH_2_O/OPLS vs TTCP/OPLS | <0.001 | *** |
| TMP∙xH_2_O/OPLS vs Cavit™ | <0.001 | *** |
| Cattiite/Na-IP6 vs Cattiite/OPLS | n.s. | – |
| Cattiite/Na-IP6 vs TTCP/OPLS | <0.01 | ** |
| Cattiite/Na-IP6 vs Cavit™ | <0.001 | *** |
| Cattiite/OPLS vs TTCP/OPLS | <0.05 | * |
| Cattiite/OPLS vs Cavit™ | <0.001 | *** |
| TTCP/OPLS vs Cavit™ | <0.001 | *** |

Table S 2: Post-hoc pairwise comparisons of shear strength values after 1 h on dentin between the different material formulations. Adjusted p-values (Tukey HSD) and corresponding significance levels are reported. n.s., not significant; *p < 0.05; **p < 0.01; ***p < 0.001; n = 12 per group.

| Comparison | p (adj.) | Significance |
| --- | --- | --- |
| TMP∙xH_2_O/Na-IP6 vs TMP∙xH_2_O/OPLS | <0.05 | * |
| TMP∙xH_2_O/Na-IP6 vs Cattiite/Na-IP6 | <0.001 | *** |
| TMP∙xH_2_O/Na-IP6 vs Cattiite/OPLS | <0.001 | *** |
| TMP∙xH_2_O/Na-IP6 vs TTCP/OPLS | <0.001 | *** |
| TMP∙xH_2_O/Na-IP6 vs Cavit™ | <0.001 | *** |
| TMP∙xH_2_O/OPLS vs Cattiite/Na-IP6 | <0.001 | *** |
| TMP∙xH_2_O/OPLS vs Cattiite/OPLS | <0.001 | *** |
| TMP∙xH_2_O/OPLS vs TTCP/OPLS | <0.01 | ** |
| TMP∙xH_2_O/OPLS vs Cavit™ | <0.001 | *** |
| Cattiite/Na-IP6 vs Cattiite/OPLS | n.s. | – |
| Cattiite/Na-IP6 vs TTCP/OPLS | n.s. | – |
| Cattiite/Na-IP6 vs Cavit™ | <0.001 | *** |
| Cattiite/OPLS vs TTCP/OPLS | n.s. | – |
| Cattiite/OPLS vs Cavit™ | <0.001 | *** |
| TTCP/OPLS vs Cavit™ | <0.001 | *** |

Table S 3: Post-hoc pairwise comparisons of shear strength values after 24 h on dentin between the different material formulations. Adjusted p-values (Tukey HSD) and corresponding significance levels are reported. n.s., not significant; *p < 0.05; **p < 0.01; ***p < 0.001; n = 12 per group.

| Comparison | p (adj.) | Significance |
| --- | --- | --- |
| TMP∙xH_2_O/Na-IP6 vs TMP∙xH_2_O/OPLS | n.s. | – |
| TMP∙xH_2_O /Na-IP6 vs Cattiite/Na-IP6 | <0.01 | ** |
| TMP∙xH_2_O /Na-IP6 vs Cattiite/OPLS | <0.001 | *** |
| TMP∙xH_2_O /Na-IP6 vs TTCP/OPLS | <0.05 | * |
| TMP∙xH_2_O /Na-IP6 vs Cavit™ | <0.001 | *** |
| TMP∙xH_2_O /OPLS vs Cattiite/Na-IP6 | <0.01 | ** |
| TMP∙xH_2_O /OPLS vs Cattiite/OPLS | <0.001 | *** |
| TMP∙xH_2_O /OPLS vs TTCP/OPLS | n.s. | – |
| TMP∙xH_2_O /OPLS vs Cavit™ | <0.001 | *** |
| Cattiite/Na-IP6 vs Cattiite/OPLS | n.s. | – |
| Cattiite/Na-IP6 vs TTCP/OPLS | n.s. | – |
| Cattiite/Na-IP6 vs Cavit™ | <0.001 | *** |
| Cattiite/OPLS vs TTCP/OPLS | n.s. | – |
| Cattiite/OPLS vs Cavit™ | <0.001 | *** |
| TTCP/OPLS vs Cavit™ | <0.001 | *** |

Table S 4: Post-hoc pairwise comparisons of shear strength values after 7 d on dentin between the different material formulations. Adjusted p-values (Tukey HSD) and corresponding significance levels are reported. n.s., not significant; *p < 0.05; **p < 0.01; ***p < 0.001; n = 12 per group.

| Comparison | p (adj.) | Significance |
| --- | --- | --- |
| TMP∙xH_2_O /Na-IP6 vs TMP∙xH_2_O /OPLS | <0.05 | * |
| TMP∙xH_2_O /Na-IP6 vs Cattiite/Na-IP6 | <0.01 | ** |
| TMP∙xH_2_O /Na-IP6 vs Cattiite/OPLS | <0.001 | *** |
| TMP∙xH_2_O /Na-IP6 vs TTCP/OPLS | n.s. | – |
| TMP∙xH_2_O /Na-IP6 vs Cavit™ | <0.001 | *** |
| TMP∙xH_2_O /OPLS vs Cattiite/Na-IP6 | <0.001 | *** |
| TMP∙xH_2_O /OPLS vs Cattiite/OPLS | <0.001 | *** |
| TMP∙xH_2_O /OPLS vs TTCP/OPLS | n.s. | – |
| TMP∙xH_2_O /OPLS vs Cavit™ | <0.001 | *** |
| Cattiite/Na-IP6 vs Cattiite/OPLS | n.s. | – |
| Cattiite/Na-IP6 vs TTCP/OPLS | <0.05 | * |
| Cattiite/Na-IP6 vs Cavit™ | <0.001 | *** |
| Cattiite/OPLS vs TTCP/OPLS | <0.05 | * |
| Cattiite/OPLS vs Cavit™ | <0.001 | *** |
| TTCP/OPLS vs Cavit™ | <0.001 | *** |

Table S 5: Post-hoc pairwise comparisons of initial shear strength values on enamel between the different material formulations. Adjusted p-values (Tukey HSD) and corresponding significance levels are reported. n.s., not significant; *p < 0.05; **p < 0.01; ***p < < 0.001; n = 12 per group.

| Comparison | p (adj.) | Significance |
| --- | --- | --- |
| TMP∙xH_2_O /Na-IP6 vs TMP∙xH_2_O /OPLS | <0.001 | *** |
| TMP∙xH_2_O /Na-IP6 vs Cattiite/Na-IP6 | <0.001 | *** |
| TMP∙xH_2_O /Na-IP6 vs Cattiite/OPLS | <0.001 | *** |
| TMP∙xH_2_O /Na-IP6 vs TTCP/OPLS | >0.05 | n.s. |
| TMP∙xH_2_O /Na-IP6 vs Cavit™ | <0.001 | *** |
| TMP∙xH_2_O /OPLS vs Cattiite/OPLS | <0.001 | *** |
| TMP∙xH_2_O /OPLS vs Cattiite/Na-IP6 | <0.001 | *** |
| TMP∙xH_2_O /OPLS vs TTCP/OPLS | <0.001 | *** |
| TMP∙xH_2_O /OPLS vs Cavit™ | <0.001 | *** |
| Cattiite/OPLS vs TTCP/OPLS | <0.001 | *** |
| Cattiite/OPLS vs Cavit™ | <0.001 | *** |
| Cattiite/Na-IP6 vs Cattiite/OPLS | <0.001 | *** |
| TTCP/OPLS vs Cavit™ | <0.001 | *** |
| Cattiite/Na-IP6 vs TTCP/OPLS | <0.001 | *** |
| Cattiite/Na-IP6 vs Cavit™ | <0.01 | ** |
|  |  |  |

Table S 6: Post-hoc pairwise comparisons of shear strength values after 1 h on enamel between the different material formulations. Adjusted p-values (Tukey HSD) and corresponding significance levels are reported. n.s., not significant; *p < 0.05; **p < 0.01; ***p < 0.001; n = 12 per group.

| Comparison | p (adj.) | Significance |
| --- | --- | --- |
| TMP∙xH_2_O /Na-IP6 vs TMP∙xH_2_O /OPLS | <0.001 | *** |
| TMP∙xH_2_O /Na-IP6 vs Cattiite/Na-IP6 | <0.001 | *** |
| TMP∙xH_2_O /Na-IP6 vs Cattiite/OPLS | <0.05 | * |
| TMP∙xH_2_O /Na-IP6 vs TTCP/OPLS | <0.001 | *** |
| TMP∙xH_2_O /Na-IP6 vs Cavit™ | <0.001 | *** |
| TMP∙xH_2_O /OPLS vs Cattiite/OPLS | <0.05 | * |
| TMP∙xH_2_O /OPLS vs Cattiite/Na-IP6 | <0.001 | *** |
| TMP∙xH_2_O /OPLS vs TTCP/OPLS | <0.001 | *** |
| TMP∙xH_2_O /OPLS vs Cavit™ | <0.001 | *** |
| Cattiite/OPLS vs TTCP/OPLS | <0.001 | *** |
| Cattiite/OPLS vs Cavit™ | <0.001 | *** |
| Cattiite/Na-IP6 vs Cattiite/OPLS | <0.001 | *** |
| TTCP/OPLS vs Cavit™ | <0.001 | *** |
| Cattiite/Na-IP6 vs TTCP/OPLS | >0.05 | n.s. |
| Cattiite/Na-IP6 vs Cavit™ | <0.001 | *** |

Table S 7: Post-hoc pairwise comparisons of shear strength values after 24 h on enamel between the different material formulations. Adjusted p-values (Tukey HSD) and corresponding significance levels are reported. n.s., not significant; *p < 0.05; **p < 0.01; ***p < 0.001; n = 12 per group.

| Comparison | p (adj.) | Significance |
| --- | --- | --- |
| TMP∙xH_2_O /Na-IP6 vs TMP∙xH_2_O /OPLS | <0.05 | * |
| TMP∙xH_2_O /Na-IP6 vs Cattiite/Na-IP6 | >0.05 | n.s. |
| TMP∙xH_2_O /Na-IP6 vs Cattiite/OPLS | >0.05 | n.s. |
| TMP∙xH_2_O /Na-IP6 vs TTCP/OPLS | >0.05 | n.s. |
| TMP∙xH_2_O /Na-IP6 vs Cavit™ | <0.001 | *** |
| TMP∙xH_2_O /OPLS vs Cattiite/Na-IP6 | >0.05 | n.s. |
| TMP∙xH_2_O /OPLS vs Cattiite/OPLS | >0.05 | n.s. |
| TMP∙xH_2_O /OPLS vs TTCP/OPLS | >0.05 | n.s. |
| TMP∙xH_2_O /OPLS vs Cavit™ | <0.001 | *** |
| Cattiite/Na-IP6 vs Cattiite/OPLS | >0.05 | n.s. |
| Cattiite/Na-IP6 vs TTCP/OPLS | <0.05 | * |
| Cattiite/Na-IP6 vs Cavit™ | <0.001 | *** |
| Cattiite/OPLS vs TTCP/OPLS | >0.05 | n.s. |
| Cattiite/OPLS vs Cavit™ | <0.001 | *** |
| TTCP/OPLS vs Cavit™ | <0.01 | ** |

Table S 8: Post-hoc pairwise comparisons of shear strength values after 7 d on enamel between the different material formulations. Adjusted p-values (Tukey HSD) and corresponding significance levels are reported. n.s., not significant; *p < 0.05; **p < 0.01; ***p < 0.001; n = 12 per group.

| Comparison | p (adj.) | Significance |
| --- | --- | --- |
| TMP∙xH_2_O /Na-IP6 vs TMP∙xH_2_O /OPLS | <0.05 | * |
| TMP∙xH_2_O /Na-IP6 vs Cattiite/Na-IP6 | <0.05 | * |
| TMP∙xH_2_O /Na-IP6 vs Cattiite/OPLS | >0.05 | n.s. |
| TMP∙xH_2_O /Na-IP6 vs TTCP/OPLS | >0.05 | n.s. |
| TMP∙xH_2_O /Na-IP6 vs Cavit™ | <0.001 | *** |
| TMP∙xH_2_O /OPLS vs Cattiite/Na-IP6 | >0.05 | n.s. |
| TMP∙xH_2_O /OPLS vs Cattiite/OPLS | >0.05 | n.s. |
| TMP∙xH_2_O /OPLS vs TTCP/OPLS | >0.05 | n.s. |
| TMP∙xH_2_O /OPLS vs Cavit™ | <0.001 | *** |
| Cattiite/Na-IP6 vs Cattiite/OPLS | >0.05 | n.s. |
| Cattiite/Na-IP6 vs TTCP/OPLS | >0.05 | n.s. |
| Cattiite/Na-IP6 vs Cavit™ | <0.001 | *** |
| Cattiite/OPLS vs TTCP/OPLS | >0.05 | n.s. |
| Cattiite/OPLS vs Cavit™ | <0.001 | *** |
| TTCP/OPLS vs Cavit™ | <0.01 | ** |

Table S 9: Post-hoc multiple comparisons of bulk penetration depth values between all tested materials using Tukey’s honestly significant difference (HSD) test. Data were analyzed following one-way ANOVA with a significance level of α = 0.05. Values represent pairwise mean differences with adjusted p-values. *p < 0.05, **p < 0.01, ***p < 0.001; n.s., not significant; n = 12 per group.

| Comparison | p (adj.) | Significance |
| --- | --- | --- |
| TMP∙xH_2_O /OPLS vs. Cavit™ | 0.705 | n. s. |
| TMP∙xH_2_O /OPLS vs. TMP∙xH_2_O /Na-IP6 | < 0.001 | *** |
| TMP∙xH_2_O /OPLS vs. Cattiite/OPLS | < 0.001 | *** |
| TMP∙xH_2_O /OPLS vs. Cattiite/Na-IP6 | < 0.001 | *** |
| Cavit™ vs. TMP∙xH_2_O /Na-IP6 | < 0.001 | *** |
| Cavit™ vs. Cattiite/OPLS | < 0.001 | *** |
| Cavit™ vs. Cattiite/Na-IP6 | < 0.001 | *** |
| TMP∙xH_2_O /Na-IP6 vs. Cattiite/OPLS | 0.823 | n. s. |
| TMP∙xH_2_O /Na-IP6 vs. Cattiite/Na-IP6 | < 0.001 | *** |
| Cattiite/OPLS vs. Cattiite/Na-IP6 | 0.021 | * |

Table S 10: Post-hoc multiple comparisons of interfacial leakage values between all tested materials using Tukey’s honestly significant difference (HSD) test. Data were analyzed following one-way ANOVA with a significance level of α = 0.05. Values represent pairwise mean differences with adjusted p-values. *p < 0.05, **p < 0.01, ***p < 0.001; n.s., not significant; n = 12 per group.

| Comparison | p (adj.) | Significance |
| --- | --- | --- |
| TMP∙xH_2_O/OPLS vs. Cavit™ | 0.006 | ** |
| TMP∙xH_2_O/Na-IP6 vs. Cavit™ | < 0.001 | *** |
| Cattiite/OPLS vs. Cavit™ | 0.506 | n. s. |
| Cattiite/Na-IP6 vs. Cavit™ | 0.169 | n. s. |
| TMP∙xH_2_O/OPLS vs. TMP∙xH2O/Na-IP6 | 0.858 | n. s. |
| TMP∙xH_2_O/OPLS vs. Cattiite/OPLS | 0.287 | n. s. |
| TMP∙xH_2_O/OPLS vs. Cattiite/Na-IP6 | < 0.001 | *** |
| TMP∙xH_2_O/Na-IP6 vs. Cattiite/OPLS | 0.034 | * |
| TMP∙xH_2_O/Na-IP6 vs. Cattiite/Na-IP6 | < 0.001 | *** |
| Cattiite/OPLS vs. Cattiite/Na-IP6 | 0.003 | ** |
